# Platelet and mitochondrial RNA is decreased in plasma-derived extracellular vesicles in women with preeclampsia—an exploratory study

**DOI:** 10.1186/s12916-023-03178-x

**Published:** 2023-11-23

**Authors:** Tove Lekva, Arvind Y.FM. Sundaram, Marie Cecilie Paasche Roland, June Åsheim, Annika E. Michelsen, Errol R. Norwitz, Pål Aukrust, Gregor D. Gilfillan, Thor Ueland

**Affiliations:** 1 Research Institute of Internal Medicine, Oslo University Hospital, Oslo, Norway; 2 Department Medical Genetics, Oslo University Hospital and University of Oslo, Oslo, Norway; 3 Department of Obstetrics, Oslo University Hospital, Oslo, Norway; 4 Faculty of Medicine, University of Oslo, Oslo, Norway; 5 Tufts University School of Medicine, Boston, MA USA; 6 Section of Clinical Immunology and Infectious Diseases, Oslo University Hospital, Oslo, Norway; 7 K. G. Jebsen Thrombosis Research and Expertise Center, University of Tromsø, Tromsø, Norway

**Keywords:** Preeclampsia, Extracellular vesicles, lncRNA

## Abstract

**Background:**

Circulating extracellular vesicles (EVs) are increased in preeclampsia (PE) and are associated with severity and progression. We examined in this exploratory cohort study if the mRNAs and long noncoding RNAs (lncRNAs) in plasma-derived EVs were dysregulated in PE compared to normal pregnancy and display different temporal patterns during gestation.

**Methods:**

We isolated EVs from plasma at weeks 22–24 and 36–38 in women with and without PE (*n*=7 in each group) and performed RNA-seq, focusing on mRNAs and lncRNAs. We validated highly expressed mitochondrial and platelet-derived RNAs discovered from central pathways in 60 women with/without PE. We examined further one of the regulated RNAs, noncoding mitochondrially encoded tRNA alanine (MT-TA), in leukocytes and plasma to investigate its biomarker potential and association with clinical markers of PE.

**Results:**

We found abundant levels of platelet-derived and mitochondrial RNAs in EVs. Expression of these RNAs were decreased and lncRNAs increased in EVs from PE compared to without PE. These findings were further validated by qPCR for mitochondrial RNAs *MT-TA, MT-ND2, MT-CYB* and platelet-derived RNAs *PPBP, PF4, CLU* in EVs. Decreased expression of mitochondrial tRNA *MT-TA* in leukocytes at 22–24 weeks was strongly associated with the subsequent development of PE.

**Conclusions:**

Platelet-derived and mitochondrial RNA were highly expressed in plasma EVs and were decreased in EVs isolated from women with PE compared to without PE. LncRNAs were mostly increased in PE. The *MT-TA* in leukocytes may be a useful biomarker for prediction and/or early detection of PE.

**Graphical Abstract:**

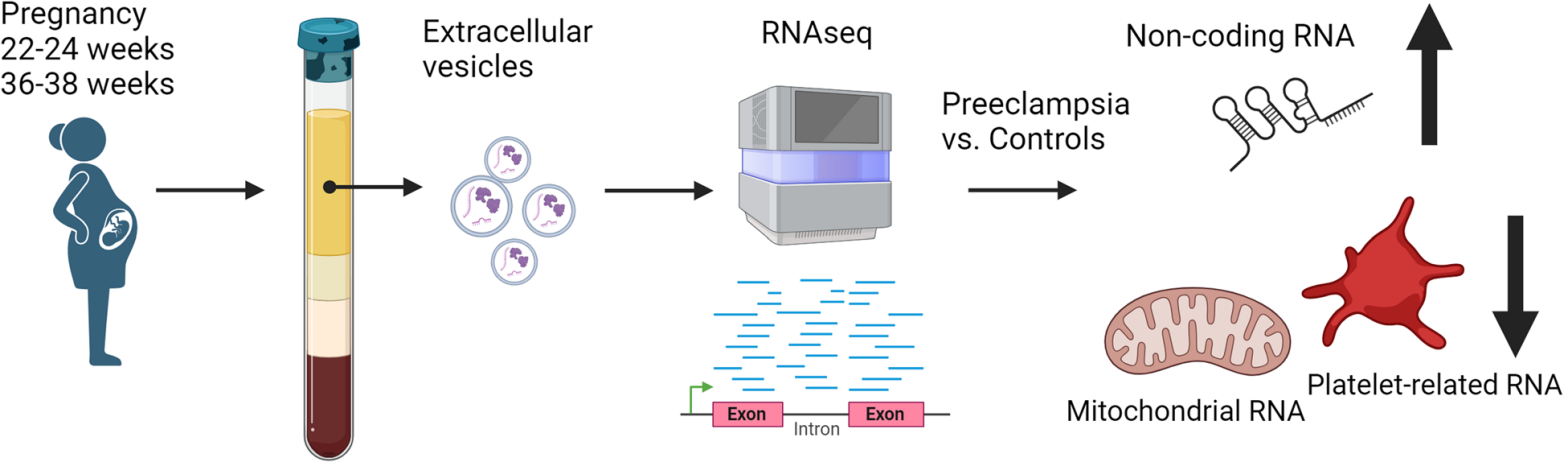

**Supplementary Information:**

The online version contains supplementary material available at 10.1186/s12916-023-03178-x.

## Background

Extracellular vesicles (EVs) play a key role in cell-to-cell communication between the mother and fetus [1, 2]. EVs have a wide range of functions such as immunological regulation and maternal vascular adaptions to pregnancy [3, 4]. The circulating concentration of EVs is increased in preeclampsia (PE) and has been associated with severity and progression of the disease [5]. EVs from women with PE and in particular exosomes may promote cytokine production from endothelial cells as well as hyperactivity of the clotting system [6]. Thus, whereas a fine-tuned function of placenta-derived vesicles, exhibiting a myriad of functions like suppression of immune reaction to the developing fetus and promoting a balanced inflammatory responses to combat infectious intruders, the EV release, their composition, and bioactivities may be harmful and used as diagnostics for pregnancy disorders [7–11].

EVs are defined as heterogeneous particles, naturally released from the cells that are delimited by a lipid bilayer and cannot replicate, including exosomes, apoptotic bodies, ectosomes or shedding microvesicles, large oncosomes, migrasomes, and exomeres [12]. These vesicles carry a rich cargo content, including lipids, proteins, RNA (mRNA, micro [mi]RNA, long noncoding RNA [lncRNA]), and DNA, that often mirrors the cell of origin. EVs in maternal blood may be derived from different sources, including various tissues, including placenta, and cells, such as platelets, endothelial cells, and various immune cells [13]. Notably, uptake of placental EVs in vitro has been demonstrated to occur in nearly every cell type [13]. Based on the protein markers identified on plasma EVs, the increased concentrations of EVs in PE seems to primarily be released by endothelial cells processing pro-coagulant properties [6].

Platelets are another important source of circulating EVs in pregnant women [6]. Pregnancy and especially PE decreases the counts of platelet-derived EVs [14] and some of the theories is that lower platelet counts in PE may contribute to lower concentrations of platelet-derived EVs or partly be due to increased trapping or participation of platelet-derived EVs in thrombin generation and fibrin clot formation or due to their association or binding with leukocytes [15]. The activation of platelets in PE is well known and EV associated P-selectin seem to reflect platelet activation in PE [16].

EVs may play important roles in determining the physiological changes during pregnancy, including formation of the fetal-maternal circulation. This process begins with extravillous trophoblast (EVT) invasion and completed around 18 weeks of gestation, being central for the remodeling of the spiral arteries, as well as preventing contact with the maternal blood flow in the intervillous space. Formation of the maternal blood flow towards the end of the first trimester occurs when cytotrophoblasts fuse to form layers of multinucleated syncytiotrophoblasts. These layers are bathed in soluble proteins and nutrients and cover the majority of the surface of the placenta. The release of EVs by EVTs in early pregnancy has been shown through the detection of soluble proteins such as human leukocyte antigen (HLA-G), which is only expressed in EVTs. Several studies have found increased circulating concentration of placental-derived EVs during PE [17, 18]. Thus, a cross-talk between the placenta and the maternal immune system is established via EVs, as placenta-derived EVs could modulate or even be incorporated by neighboring and distant maternal immune cells, while EVs produced by maternal immune and endothelial cells could modulate placental responses [3, 4, 19, 20]. During early stages of pregnancy, EVs derived from endometrium and trophoblasts can influence the maternal immune response, whereas towards the advanced stages of gestation, the EVs released by the placenta, mainly syncytiotrophoblast-derived EVs, are the main players [4, 21]. The molecular cargo of syncytiotrophoblasts varies in PE pregnancies, but an augmented expression of tissue factor, endoglin and fms-like tyrosine kinase (Flt-1) which are key mediators of pathological response in PE has been reported in EVs [22].

PE is a pregnancy-specific hypertensive syndrome, which seriously threatens the safety of mother and infant. However, there is still no accurate early biomarker for the diagnosis of preeclampsia, and its etiology and pathogenesis have not been fully elucidated. Previous studies have shown that a large number of differently expressed lncRNA are present in the placental tissue in PE [23, 24] potentially playing a vital role in the pathogenesis of PE [25, 26], but these issues are far from clear. While several studies have investigated the role of circulating miRNAs in PE [27], studies on the RNA cargo, and especially lncRNA, within plasma EVs in the setting of PE are scarce or lacking.

In this exploratory study, we examined if the mRNAs and lncRNAs in plasma EVs are dysregulated in PE compared to normotensive pregnancies, processing immune-modulatory effects on the maternal immune system that could be used for prediction of PE development, as well as progression. All examinations were performed in the previously described STORK study, a prospective longitudinal cohort study [28]. From this cohort, we isolated EVs from plasma at weeks 22–24 and 36–38 in women with and without PE, and performed RNA-seq on selected samples (discovery cohort). We classified the RNAs, focusing on mRNAs and lncRNAs, and investigated which biological pathways they were involved in. We then validated highly expressed RNAs in EVs from a separate subsample of 60 women with/without PE. Finally, we assessed one of the regulated RNA, the noncoding mitochondrially encoded tRNA alanine (MT-TA), in leukocytes (PBMC) and plasma to investigate its biomarker potential and association with clinical markers of PE pathology.

## Material and methods

The STORK study, a prospective longitudinal cohort study in which 1031 women of Scandinavian heritage with low-risk singleton pregnancies who gave birth at Oslo University Hospital, Rikshospitalet, Oslo, Norway, between 2002 and 2008, were followed throughout pregnancy [28]. Exclusion criteria included the presence of one or more severe chronic diseases (such as pre-gestational diabetes, lung, cardiac, gastrointestinal, and/or renal disease). None of the included individuals had any symptoms of acute infections and none were using antibiotics or anti-viral agents when included into the study, although asymptomatic infection cannot be excluded. Each woman had four study-related antenatal visits at 14–16, 22–24, 30–32, and 36–38 weeks’ gestation. In the current study, additional exclusion criteria included gestational diabetes mellitus. A flow chart showing inclusion and sample collection is shown in Additional file 1: Fig. S1. We included 7 women with PE and 7 age- and BMI-matched women without PE in the RNA-seq discovery experiment of plasma EVs and 28 women with PE and 32 women without PE in the validation of some of the differentially expressed (DE) RNAs in EVs. Patients from the discovery study were excluded in the EV validation study. For the DE RNAs in leukocytes, we used samples from 38 women with PE and 215 women without PE and in plasma 35 women with PE and 35 women without PE (Table 1). These samples were chosen from a sub-study including 38 women with PE and 215 normal pregnancies, described and published previously [29]. For PE women, we used all available samples in our study while control samples in the EV validation study and for direct isolation from plasma were randomly selected.

**Table 1:** Clinical and demographic characteristics of the study population selected from the STORK cohort

**Criteria**	**RNA-seq**			**EV validation**			**Plasma validation**			**Leukocyte validation**		
	**NP** (*n*=7)	**Preeclampsia** (*n*=7)	***P*-value**	**NP** (*n*=32)	**Preeclampsia** (*n*=28)	***P*-value**	**NP** (*n*=35)	**Preeclampsia** (*n*=35)	***P*-value**	**NP** (*n*=215)	**Preeclampsia** (*n*=38)	***P*-value**
Age (years)	29 ± 3	29 ± 4	0.653	32 ± 4	30 ± 3	**0.037**	32 ± 4	30 ± 4	**0.019**	32 ± 4	30 ± 4	**0.001**
Gestational age at delivery (week)	40 ± 1	39 ± 2	0.228	40 ± 1	39 ± 3	**0.002**	40 ± 1	39 ± 3	**0.001**	40 ± 1	39 ± 3	**<0.001**
Multiparous n (%)	3 (43)	2 (29)	0.577	15 (47)	8 (29)	0.187	16 (46)	10 (29)	0.138	110 (47)	11 (29)	**0.035**
Uterine artery PI												
22–24 weeks	0.74 (0.52, 1.06)	1.05 (0.80, 1.92)	0.151	0.79 (0.72, 0.94)	0.99 (0.79, 1.16)	**0.045**	0.81 (0.70, 0.97)	1.05 (0.79, 1.17)	**0.006**	0.83 (0.72, 0.99)	1.01 (0.79, 1.17)	**0.007**
36–38 weeks	0.81 (0.68, 1.24)	0.87 (0.73, 1.03)	1.000	0.72 (0.60, 0.86)	0.84 (0.68, 1.09)	0.060	0.72 (0.59, 0.85)	0.84 (0.69, 1.08)	**0.021**	0.72 (0.60, 0.84)	0.84 (0.69, 1.08)	**0.007**
BMI (kg/m^2^)												
14–16 weeks	23.2 (21.2, 23.6)	23.1 (20.4, 26.6)	0.668	23.9 (21.2, 26.8)	28.1 (24.2, 30.6)	**0.002**	23.6 (21.2, 26.4)	27.5 (22.5, 29.8)	**0.007**	23.5 (21.3, 25.4)	27.4 (23.1, 29.9)	**<0.001**
22–24 weeks	23.8 (22.4, 25.1)	23.8 (21.7, 26.6)	0.886	24.9 (22.4, 27.7)	29.5 (25.4, 32.7)	**0.002**	24.8 (22.4, 27.6)	28.1 (23.2, 31.9)	**0.011**	24.8 (22.5, 26.8)	29.0 (23.8, 32.1)	**<0.001**
30–32 weeks	25.0 (23.4, 25.8)	25.1 (23.4, 28.3)	0.886	27.2 (23.9, 28.8)	31.7 (28.5, 33.4)	**<0.001**	26.4 (23.8, 28.8)	31.3 (25.6, 33.3)	**0.004**	26.1 (23.7, 28.3)	31.2 (26.0, 33.3)	**<0.001**
36–38 weeks	26.1 (24.0, 26.7)	27.6 (24.1, 29.7)	0.199	27.8 (25.2, 30.0)	33.7 (28.2, 35.0)	**0.001**	27.4 (25.1, 30.0)	31.8 (27.1, 34.8)	**0.006**	27.2 (24.8, 29.5)	32.2 (27.3, 34.8)	**<0.001**
Systolic BP (mmHg)												
14.16 weeks	110 (110, 120)	110 (109, 120)	0.836	110 (105, 116)	115 (110, 120)	**0.020**	110 (105, 115)	115 (110, 120)	**0.013**	110 (100, 119)	115 (110, 120)	**0.002**
22–24 weeks	110 (110, 120)	110 (104, 121)	0.836	110 (103, 119)	120 (104, 120)	**0.136**	110 (102, 115)	118 (105, 120)	0.119	110 (100, 115)	115 (104, 120)	**0.046**
30–32 weeks	120 (105, 125)	110 (105, 120)	0.620	110 (106, 120)	120 (110, 130)	**0.064**	110 (105, 120)	120 (110, 125)	**0.102**	110 (105, 120)	120 (110, 126)	**0.002**
36–38 weeks	118 (111, 121)	125 (120, 150)	0.101	115 (100, 120)	130 (118, 138)	**<0.001**	115 (100, 120)	130 (120, 139)	**<0.001**	110 (105, 120)	130 (120, 138)	**<0.001**
Diastolic BP (mmHg)												
14.16 weeks	65 (60, 75)	70 (69, 73)	0.366	68 (60, 70)	70 (65, 80)	**0.062**	65 (60, 70)	70 (65, 79)	**0.016**	70 (60, 70)	70 (65, 80)	**0.004**
22–24 weeks	70 (60, 70)	70 (60, 80)	0.836	65 (60, 70)	70 (60, 80)	**0.035**	75 (60, 70)	70 (60, 80)	**0.020**	65 (60, 70)	70 (60, 80)	**0.001**
30–32 weeks	70 (60, 80)	70 (65, 80)	0.902	70 (60, 75)	75 (70, 84)	**0.011**	70 (60, 75)	70 (65, 80)	**0.010**	70 (60, 70)	73 (70, 80)	**<0.001**
36–38 weeks	73 (68, 80)	80 (70, 95)	0.181	70 (65, 80)	90 (78, 93)	**<0.001**	70 (65, 80)	85 (76, 94)	**<0.001**	70 (69, 80)	85 (78, 93)	**<0.001**
MAP (mmHg)												
14–16 weeks	80 (77, 90)	83 (82, 88)	0.471	81 (76, 85)	86 (80, 90)	**0.018**	80 (75, 85)	84 (80, 90)	**0.005**	80 (75, 85)	85 (80, 90)	**<0.001**
22–24 weeks	83 (77, 87)	84 (75, 93)	0.999	80 (77, 86)	85 (80, 93)	**0.026**	80 (77, 85)	85 (79, 93)	**0.016**	80 (73, 85)	83 (79, 93)	**0.001**
30–32 weeks	83 (77, 95)	83 (77, 93)	0.949	83 (77, 90)	89 (82, 99)	**0.018**	83 (77, 90)	87 (82, 95)	**0.019**	82 (77, 87)	87 (83, 95)	**<0.001**
36–38 weeks	88 (82, 94)	95 (87, 113)	0.132	84 (80, 92)	103 (90,108)	**<0.001**	84 (79, 92)	100 (90, 108)	**<0.001**	85 (80, 93)	100 (90, 108)	**<0.001**

Data given as mean ± SD when normally distributed and median (25th, 75th percentile) when not normally distributed

*NP* Normal pregnancy, *EV*, Extracellular vesicles, *BMI* Body mass index, *MAP* Mean arterial pressure, *BP* Blood pressure, *PI* Pulsatility index

### Preeclampsia

PE was diagnosed by new-onset hypertension (sustained elevation in BP ≥140/90 mmHg) and significant proteinuria (urinary total protein/creatinine ratio >30 mg/mmol or +1 on urine dipstick) using old criteria since the cohort was collected between 2002 and 2008. Almost all women developing PE were diagnosed after 34 weeks’ gestation (only 3 women before week 34). No women were treated with aspirin as this recommendation was not yet implemented in Norway during the period of inclusion (2002–2008).

### Uterine artery pulsatility index

The ultrasound examinations were done using the same equipment for all participants (Acuson Aspen, Mountain View, CA, USA). The mean uterine artery pulsatility index (PI) was obtained by bilateral Doppler flow velocity measurements using an abdominal approach. The measurements were performed close to the crossing of the external iliac arteries. The insonation angle was as low as possible. Pulsatility index (PI) is defined as the difference between the peak systolic flow and minimum diastolic flow velocity, divided by the mean velocity recorded throughout the cardiac cycle. The formula for PI = (peak systolic velocity − end diastolic velocity)/mean flow velocity. The pulsatility index was calculated as the mean of three heart cycles on each side. For each woman, we used the mean values of the right and left side. Some of the uterine artery PI data was missing (approximately 10 PE and 40 normal pregnancy in the largest validation (leukocyte) cohort).

### Collection, storage, and RNA extraction of EVs

Whole blood (8 ml) was drawn (after an overnight fast) directly into BD Vacutainer CPT Tubes (Becton Dickinson Vacutainer Systems, Franklin Lakes, NJ, USA) with sodium citrate additives at weeks 22–24 and 36–38. The tubes were centrifuged at room temperature in a horizontal rotor (swing-out head) for 20 min at 1800 RCF (Relative Centrifugal Force) with the use of a FICOLL™ Hypaque™ gradient centrifugation and maternal plasma were collected from the top and above the mononucleated cell layer and stored at −80 °C. The plasma was not depleted for platelets before EV isolation. The unthawed plasma stored at −80 °C was thawed at room temperature centrifuged at 3000*g* for 5 min (to eliminate residual cellular material, including thrombocyte fragments, but still retain the vast majority of EVs) and total RNA from EVs was isolated from 800µL plasma using the exoRneasy serum/plasma kit (Qiagen), as previously published [29]. Briefly, the total procedure for isolating RNA from EVs consisted of two phases: EV purification and RNA isolation. In the EV purification stage, precentrifuged sample was mixed with Buffer XBP and bound to an exoEasy membrane affinity spin column (500*g*, 1min). The bound EVs was washed with Buffer XWP (5000*g*, 5 min), and then lysed with QIAzol (5000g, 5 min). In the RNA extraction step, chloroform was added to the QIAzol eluate, and the aqueous phase was recovered and mixed with ethanol. Total RNA binds to the spin column, where it was washed three times and eluted. The RNA yield was from 100 to 2000ng (median 340ng) in 20 µl total. Purity and concentration of isolated total RNA was measured using Nanodrop ND-1000 Spectrophotometer (Thermo Fisher Scientific Inc., USA) and RNA integrity was investigated using Agilent 2100 Bioanalyzer (Agilent Technologies, USA). When investigating the RNA yield of the EVs in the plasma we found the RNA yield to be lower at weeks 22–24 in the PE samples compared to controls; however, no difference at weeks 36–38 between the groups were found.

### RNA-seq and data analysis

The samples chosen for RNA-seq were matched on maternal age and BMI between normal pregnancy and PE groups (*n*=7 in each group). To remove residual DNA in the samples, they were first digested with Heat&Run DNase (ArticZymes Technologies, Tromsø, Norway) according to manufacturer`s instruction. Sequencing libraries were prepared from 10 ng of total RNA using the SMARTer Stranded Total RNA-seq Kit v2-Pico Input Mammalian (Takara Bio, San Jose, CA, USA), according to the manufacturer’s instructions, without fragmentation (with or without fragmentation were tested in two samples before analysis of all the samples) and employed 16 cycles PCR amplification. Libraries were indexed with UDI indexes from the SMARTer RNA UDI set A set (Takara). The libraries were sequenced together on an SP flowcell, with 150 bp paired-end sequencing on a NovaSeq 6000 instrument (Illumina, San Diego, CA, USA). Reads containing adapter sequences and low-quality reads were trimmed/removed using BBDuk (part of BBMap v34.56; parameters: ktrim=r k=23 mink=11 hdist=1 tbo tpe qtrim=r trimq=15 maq=15 minlen=36 forcetrimright=149) [30]. Clean reads in fastq format were aligned against the ENSEMBL Human GRCh38 release 104 genome using HISAT2 v2.1.0 (parmater: --rna-strandness RF) [31]. Reads mapping to the genes were counted using featureCounts v1.4.6-p1 (parameter: -p -s 2) [32]. DE genes in the RNA-seq data were calculated by DESeq2 V1.34.0 using recommended default settings/parameters by the tool authors [33] using SARTools v1.7.4 R v4.1.1 packages (Additional file 1: Table S1) [34]. Outlier detection (Cook distance cut-off) and filtering out low expressed genes was performed using the default method in DESeq2. Gene set enrichment analysis among the significantly DE genes against the gene ontology (GO) and KEGG databases were performed using the web-based g:Profiler (https://biit.cs.ut.ee/gprofiler/gost) [35]. We included up to 14 main pathways (significantly) in each of the source programs in the Additional file 1: Fig.S2 and Fig.S3, while all significantly pathways are included in the Additional file 2.

### Collection, storage, and total RNA extraction from plasma

Whole blood was drawn in CPT tubes and plasma collected and stored as described above. Total RNA was isolated from 200µL plasma using the miRneasy serum/plasma kit (Qiagen). The spike-in control utilized *C. elegans* miR-39 miRNA was used based on recommendation from the kit instruction. After addition of QIAzol lysis reagent, 3.5 µl of 1.6×10^8 copies/µl of mir-39 was added to the mix of QIAzol and plasma. The RNA yield was from 300ng to 4µg (median 900ng) in 20 µl total.

### Collection, storage, and RNA extraction of maternal leukocytes

Whole blood was drawn in CPT tubes as described above, and leukocytes (peripheral blood mononuclear cells, PBMC) were collected by pipetting the mononucleated cell layer and stored at −80 °C. RNA was extracted using Magnapure Isolation Kit (Roche Life Science, Penzberg, Germany) at weeks 22–24 and Magmax Isolation Kit (Applied Biosystems, Carlsbad, CA) at weeks 36–38, due to change in instruments at the laboratory, as previously published [36]. Both automated methods used magnetic-bead technology to extract RNA. The RNA yield was from 400ng to 10µg (median 1500ng) in 50 µl total.

### Quantitative real-time polymerase chain reaction

Reverse transcription was performed using High Capacity cDNA Archive Kit (Applied Biosystems, Foster City, CA) for leukocytes RNA (100ng RNA input), RT^2^ First Strand Kit (Qiagen for EV RNA) (150ng RNA input), and miScript II RT Kit (Qiagen) for RNA from plasma (Qiagen) (400ng RNA input). RNA quantification was performed using SYBR Green PCR Fast Mix (Quantabio, Beverly, MA) for leukocytes, RT2 lncRNA SYBR Green qPCR kit (Qiagen) for EVs, and miScript SYBR Green PCR Kit (Qiagen) using the standard curve method on an ABI Prism 7900 (Applied Biosystems). Primers for Ce_miR-39_1 miScript Primer Assay were from Qiagen. Sequence-specific intron spanning oligonucleotide primers for *PF4, PPBP, MT-TA, MT-CYB, MT-ND2, RPLP0, GAPDH* and *ACTB* were designed by NCBI Primer Blast (Additional file 1: Table S2). The miRNAs mir-515-p (cat.no.YP00204431) and mir-518b (cat.no.YP00204405) primers were bought from Qiagen (Netherlands). Transcript expression levels were normalized to *ACTB* and *GAPDH* and expressed as relative RNA levels in leukocytes, miR-39 for plasma-derived RNA, and *RPLP0* in EVs (ACTB and GAPDH were regulated between PE and normal pregnancy when investigating the RNA-seq data from EVs).

### Biochemical analysis

Peripheral venous blood was drawn in the morning between 07:30 and 08:30AM after an overnight fast, collected in tubes with citrate additives, centrifuged for 25 min at 3000*g* at 4°C, separated, and stored at −80°C until analysis. Levels of P-selectin (catalog# DY137), PF4 (catalog#DY795), sFlt1 (catalog# DY321B) and PlGF (catalog#DY264) were measured in duplicate by enzyme immuno-assay with antibodies obtained from R&D Systems at weeks 14–16, 22–24, 30–32 and 36–38. The markers were analyzed in a set-up that combined a CyBi SELMA (CyBio, Jena, Germany), an EL406washer/dispenser (Biotek, Winooski, VT), and a Synergy H1 microplate reader (Biotek).

### Statistical analysis

Data are expressed as mean ± SD when normally distributed and median (25th, 75th percentile) when skewed. Comparison of demographics between women with/without PE was performed using *t*-test or Mann-Whitney *U* test, depending on distribution, and chi-square test for categorical variables. Temporal changes in EV RNA expression, leukocytes, and plasma during pregnancy between normal pregnancy and women with PE were assessed using linear mixed model analysis with subject as random effect and time and PE diagnosis as fixed effects (also as interaction) adjusted for age and BMI 14–16 weeks (considered pre-pregnancy). Associations at individual time points within normal pregnancy and PE groups were also assessed by Spearman correlation analysis. The discriminatory properties of RNAs in EV and leukocytes at 22–24 weeks and subsequent development of PE was assessed by ROC analysis. Cut-offs were determined using Youden’s index and we specify likelihood ratios (LHR) based on these. Two-tailed *p*-values <0.05 were considered significant. Statistical analyses were conducted using SPSS for Windows, version 28.0 (Chicago, IL, USA). A gene in RNA-seq was considered as differently expressed when the false discovery rate (FDR) was <0.1. FDR was calculated according to Benjamini and Hochberg.

## Results

### Study population

The clinical and demographic characteristics of the study cohorts are presented in Table 1. In the discovery population (*n*=7 in each group), where the groups were matched, there were no significant differences in demographics between the PE and normotensive populations. In the EV validation cohort (*n*=28 PE and 32 controls, excluding the 7 from the discovery group), women with PE were younger, had lower gestational age at delivery, and higher uterine artery PI (Table 1). The same differences were seen in the plasma/leukocyte cohort (normal pregnancy, *n*=35/*n*=215; PE, *n*=35/*n*=38, respectively); in addition, more multiparous women were present in the normal pregnancy group. Moreover, women with PE had higher BMI, systolic blood pressure, diastolic blood pressure and MAP throughout pregnancy, except in the discovery (i.e. RNA-seq).

### Platelet and mitochondria derived RNA is most abundantly expressed RNAs in EVs based on RNA-seq analysis

**Fig. 1:**
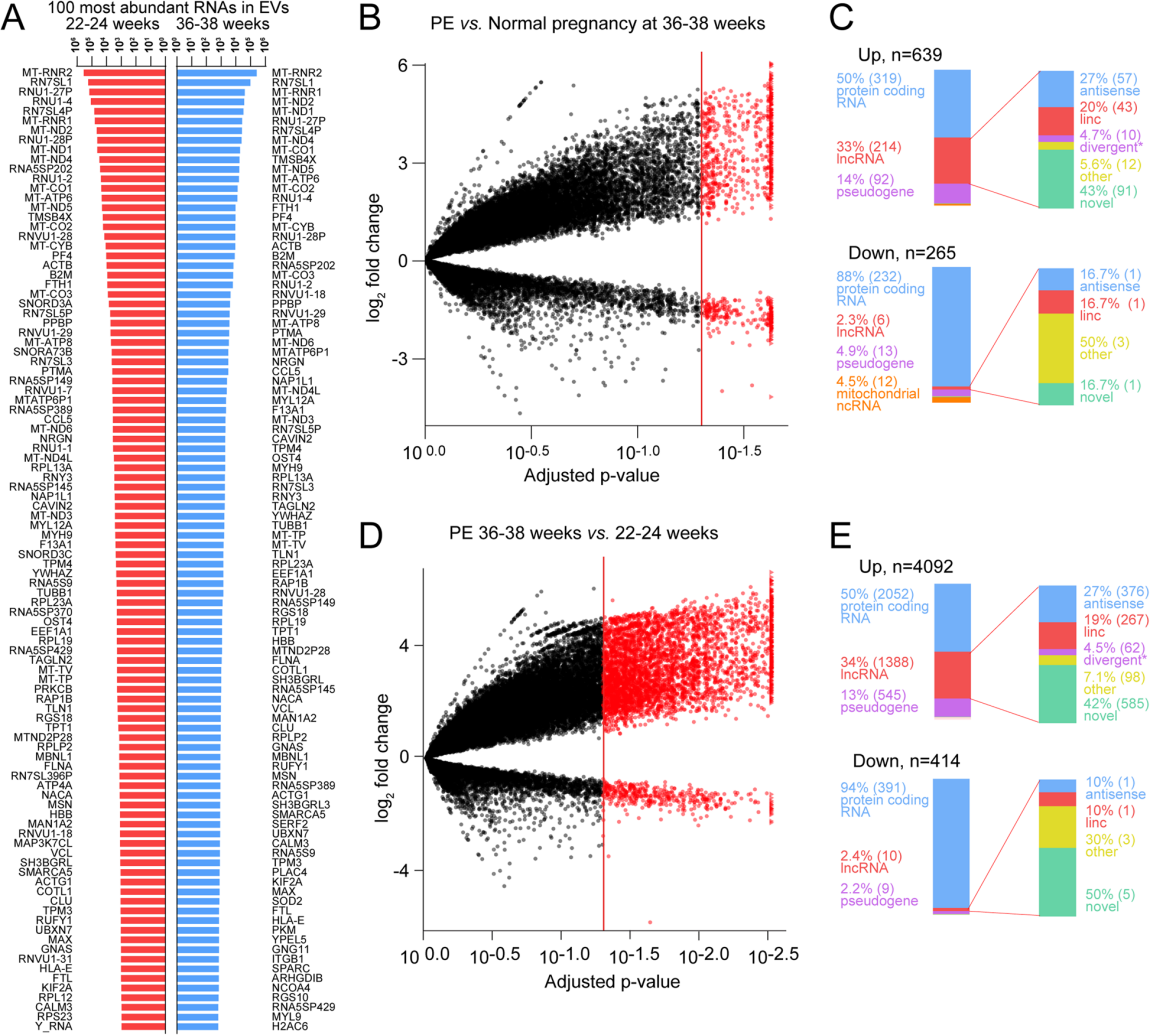
**A** The 100 most abundant RNAs in plasma EVs in normotensive controls (*n*=7) from the RNA-seq analysis at 22–24 weeks and 36–38 weeks gestation. **B** Volcano plot and distribution of differentially expressed RNAs from plasma extracellular vesicles are shown between women with preeclampsia (PE) (*n*=7) and normotensive controls (*n*=7) at gestational weeks 36–38 (**B**), and between gestational weeks 22–24 and 36–38 in women who subsequently developed PE (**D**). The distribution (number and percentage) of the different classes of differentially regulated RNA and lncRNA are shown between PE and normotensive pregnancies (**C**) and between gestational weeks 22–24 and 36–38 in women who subsequently developed PE (**E**). *divergent/overlapping/intronic

EVs carry a rich cargo that mirrors the cell of origin. Investigating the 100 most abundant RNAs in normal pregnancies (Fig. 1A), we found some patterns. RNAs identified in a recent study of healthy blood donors to be a signature for platelets (including *B2M, PPBP, TMSB4X, ACTB, FTL, CLU, PF4, F13A1, GNAS, SPARC, PTMA, TAGLN2,* and *OST4*) [37] were found in the top 100 expressed RNAs in our RNA-seq data, suggesting that platelets are a major source of plasma EVs during normal pregnancy. Mitochondrial RNAs, both noncoding (*MT-RNR1,2*) and mRNA (*MT-ND1,2,4,5, MT-CO1,2,3, MT-CYB, MT-ATP6,8*), were also highly expressed in EVs.

### Differentially expressed RNAs identified in the RNA-seq analysis

We next compared RNA expression between and within the PE and control groups in the discovery population. Using RNA-seq analysis, we found 904 DE RNAs between PE and normal pregnancy at weeks 36–38 (FDR<0.1) (shown in the volcano plot, Fig. 1B). Of these, 265 were significantly downregulated and 639 were significantly upregulated in PE (Fig. 1C). Classifying these DE RNAs into groups based on biological processes revealed a large amount of lncRNA where 2.3 % (*n*=6) were downregulated and 33% (*n*=214) were upregulated in PE. No DE (FDR<0.1) RNAs at 22–24 weeks were detected between women with PE and normal pregnancy.

When assessing DE RNAs (FDR<0.1) from weeks 22–24 to 36–38 in women with PE, we found 4506 DE RNAs (FDR<0.1) (shown in the volcano plot, Fig. 1D). Of 414 significantly downregulated and 4092 significantly upregulated RNA (Fig. 1E), 2.4% (*n*=10) and 33.9% (*n*=1388) were lncRNA, respectively. No changes were observed in the normal pregnancy group from 22–24 to 36–38 weeks.

The lncRNA can be further classified as novel followed by antisense, long intergenic nc (linc), other, and divergent /overlapping /intronic ncRNA. Most of the lncRNA were expressed at low levels (the most abundant lncRNA was expressed at approximately the same level as the lowest of the 100 most abundant RNA [Fig. 1A]).

### Differentially expressed RNAs from platelet-derived EVs and mitochondrial RNA

**Fig. 2:**
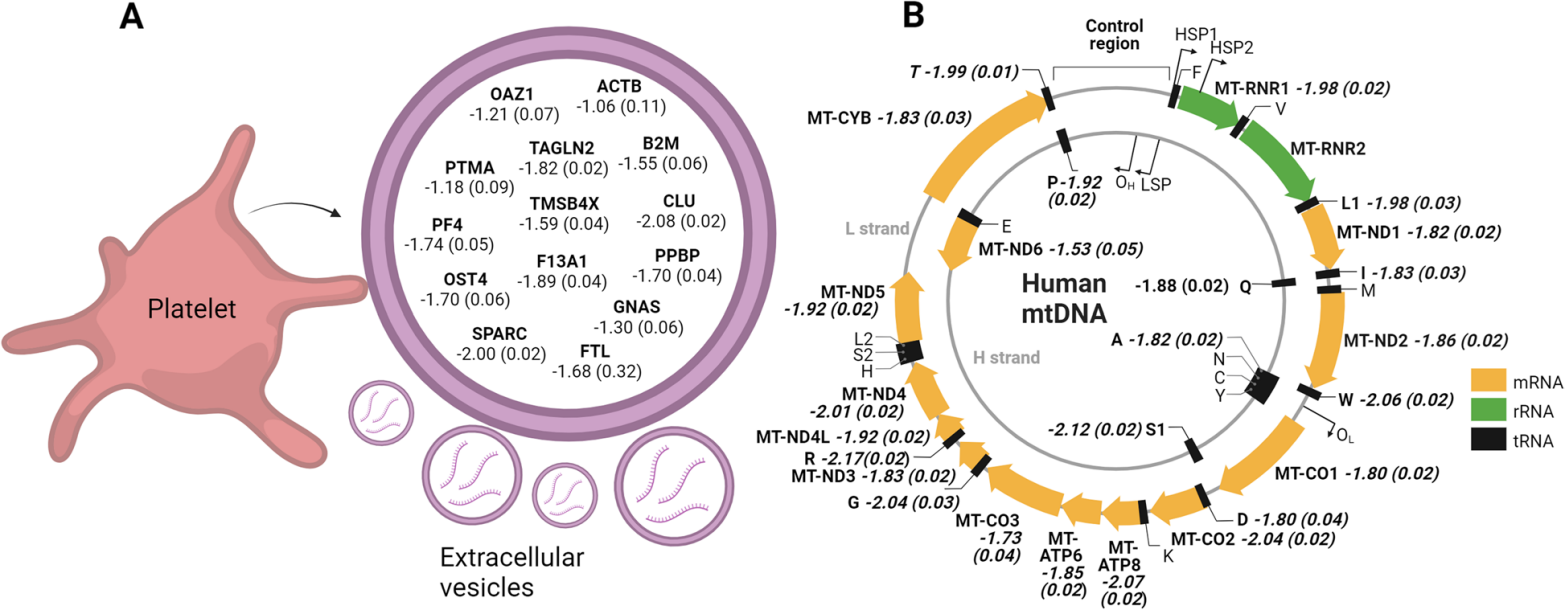
Differentially regulated RNAs at 36–38 weeks between women with PE and controls from platelet-derived extracellular vesicles (**A**) and mitochondria (**B**) as characterized by the RNA-seq analysis. Numbers are log-2 fold change (adjusted *p*-value) in PE vs. normal pregnancy samples. Created with BioRender.com

To further explore the RNA-seq data, we characterized them in relation potential sources. We found that RNAs known to be a signature of platelets (*PF4, PPBP, TMSB4X, F13A1, TAGLN2, CLU* and *SPARC*) [37] were present at significantly lower levels in EVs from PE compared to normal pregnancy at 36–38 weeks (Fig. 2A). The integrins *ITGA2B*, *ITGB3* and chemokine *CCL5*, known to be platelet-derived, were also significantly decreased in EVs from PE compared to normal pregnancy (log2fold −2.3, −2.2, and −1.7; FDR adjusted *p*=0.014, *p*=0.024, and *p*=0.048, respectively). Mitochondrial RNA was also abundant and downregulated in PE compared to normal pregnancy based on the RNA-seq analysis at 36–38 weeks. Of the mitochondrial RNAs, we found 13 mRNA (*MT-ND1-6, MT-ND4L, MT-CYB, MT-CO1-3, MT-ATP6,8*), 1 rRNA (*MT-RNR1*) and 11 tRNA (*MT-TA, MT-TL1, MT-TI, MT-TW, MT-TD, MT-TG, MT-TT, MT-TR, MT-TP, MT-TS1, MT-TQ*) significantly lower in EVs from PE compared to normal pregnancy (Fig. 2B). Evaluating the DE RNAs from weeks 22–24 to 36–38 in women who subsequently developed PE showed that most of the platelet-derived RNAs significantly decreased during that time period (Additional file 1: Fig. S4) as did some of the mitochondrial RNAs (Additional file 1: Fig. S5).

### Validation of some differentially expressed RNAs using qPCR

**Fig. 3:**
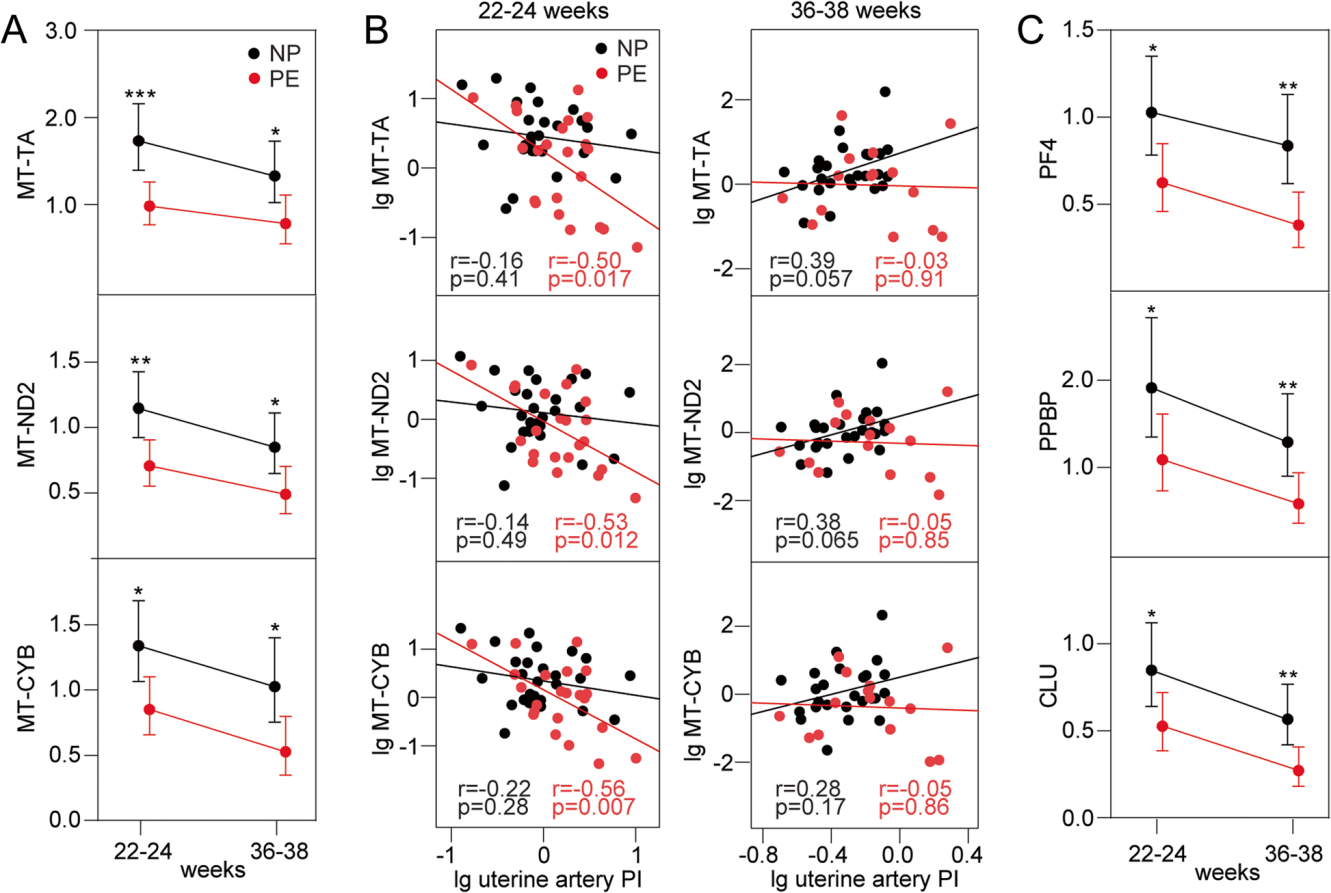
PCR validation of data from the EV RNA-seq analysis and their association with uterine artery doppler pulsatility index (PI). Samples from women with preeclampsia (PE)/normal pregnancy (NP), *n*=28/*n*=32 at 22–24 weeks and *n*=20/*n*=32 at 36–38 weeks, respectively, were used. **A** Validation of the mitochondrial RNAs *MT-TA*, *MT-ND2* and *MT-CYB* at weeks 22–24 and 36–38 between women who subsequently developed PE and controls (NP), adjusted for age and BMI. **B** Scatter plot showing the correlation between mitochondrial RNAs and uterine artery PI at weeks 22–24 and 36–38 between women who subsequently developed PE and controls (NP). **C** Validation of the platelet-derived RNAs *PF4, PPBP* and *CLU* at weeks 22–24 and 36–38 between women who subsequently developed PE and controls (NP), adjusted for age and BMI. *r*=correlation coefficient, *p-value<0.05, ** *p*<0.01, ***p<0.001

We next validated selected transcripts from the discovery RNA-seq data in the remaining PE women and randomly selected controls (*n*=60, Table 1, EV validation). We chose transcripts that were abundantly expressed and were differently expressed between PE and controls at weeks 36–38. As shown in Fig. 3A, we confirmed that mitochondrial tRNA *MT-TA* as well as the mRNAs *MT-CYB* and *MT-ND2* were significantly downregulated in PE (at weeks 22–24) prior to diagnosis and decreased significantly further as gestational age progressed.

Alterations in waveforms in the uterine artery as reflected by Doppler indices are associated with the increased risk of developing PE [38]. As shown in Fig. 3B, mitochondrial tRNAs were significantly negatively correlated with uterine artery PI in women with PE, but not in normal pregnancy, at 22–24 weeks. Conversely, these mitochondrial tRNAs were positively correlated, however not significant, with uterine artery PI in normal pregnancy at 36–38 weeks. No correlation was seen at this time point in PE.

The platelet-derived mRNAs *PF4*, *PPBP*, and *CLU* showed a similar pattern as the mitochondrial tRNAs (i.e. they were significantly decreased in women with PE before the diagnosis and decreased significantly during gestation) (Fig. 3C), but were not correlated with uterine artery PI, except for *CLU*, *PPBP* and *PF4* in PE at 22–24 weeks (PF4 normal pregnancy; correlation coefficients 22–24 weeks *r*=−0.16 *p*=0.44, 36–38 weeks *r*=0.17 *p*=0.413 and PE 22–24 weeks *r*=−0.43 *p*=0.047, 36–38 weeks *r*=−0.08 *p*=0.782, *PPBP* normal pregnancy; correlation coefficients 22–24 weeks *r*=−0.09 *p*=0.662, 36–38 weeks *r*=0.01 *p*=0.975, and PE 22–24 weeks *r*=−0.49 *p*=0.021, 36–38 weeks *r*=0.05 *p*=0.849, *CLU* normal pregnancy; correlation coefficients 22–24 weeks *r*=−0.14 *p*=0.493, 36–38 weeks *r*=0.18 *p*=0.404 and PE 22–24 weeks *r*=−0.47 *p*=0.026, 36–38 weeks *r*=0.06 *p*=0.832).

### Assessment of discriminatory abilities of RNAs

**Fig. 4:**
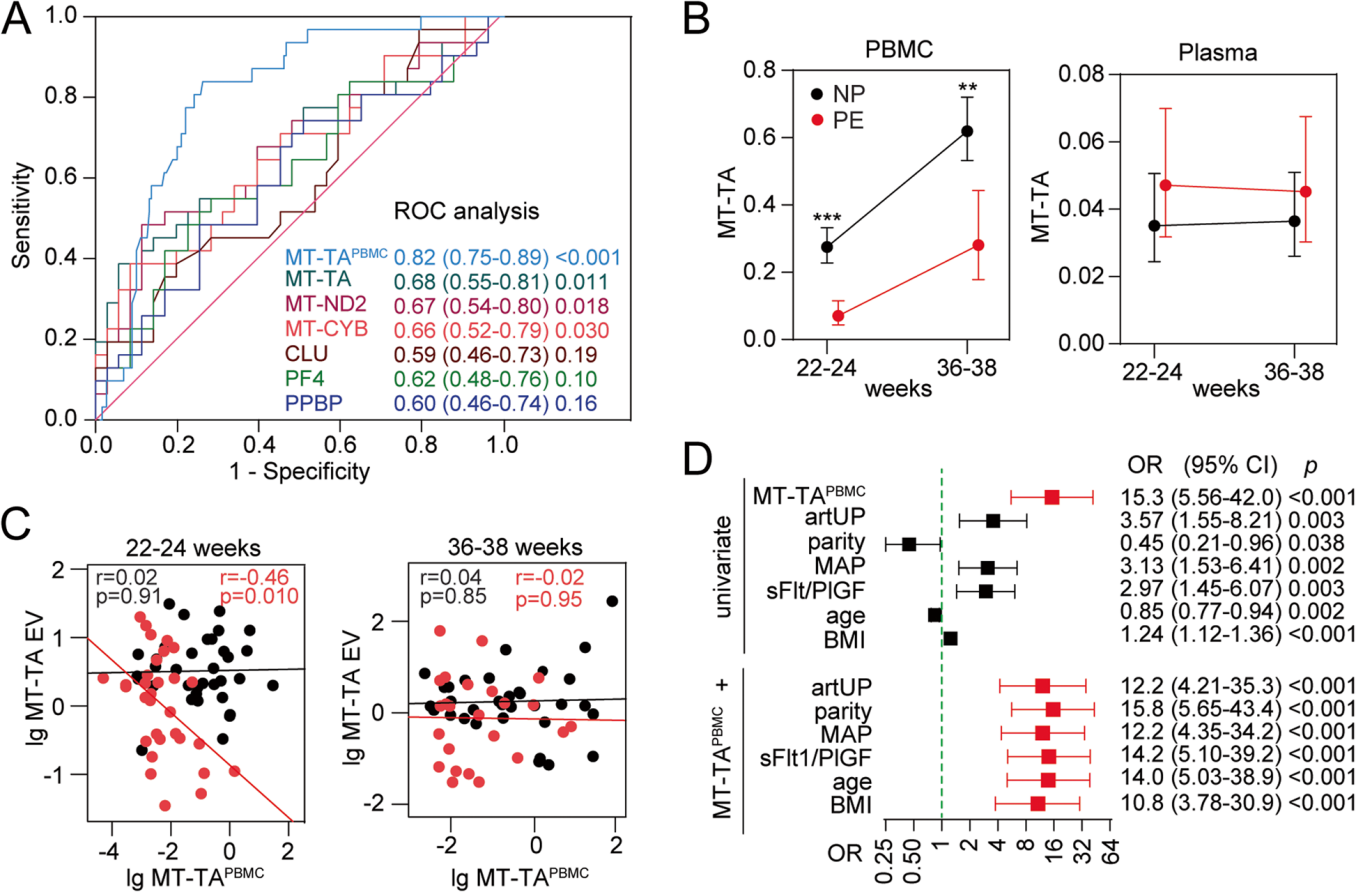
**A** ROC analysis to identify the best predictor of preeclampsia (PE) at 22–24 weeks using RNAs from EVs *MT-TA*, *MT-ND2*, *MT-CYB*, *CLU*, *PF4* and *PPBP* (35 PE, 35 normal pregnancy (NP)) and *MT-TA*^PBMC^ from leukocytes (peripheral blood mononuclear cells, PBMC) (38 PE, 215 NP). **B** Expression of mitochondrial tRNA *MT-TA* in leukocytes (PBMC) and plasma between women who subsequently developed PE and those that did not (NP) at weeks 22–24 and 36–38, adjusted for age and BMI. **C** Scatter plot showing correlation between *MT-TA* in leukocytes (PBMC) and EVs at weeks 22–24 and 36–38 between women who developed PE and those that did not (NP). *r*=correlation coefficient, *p<*, ** *p*<0.01, ***p<0.001. **D** Logistic regression analysis of predictors of PE at 22–24 weeks. The top part shows MT-TA^PBMC^ and established risk predictors of PE in univariate analysis. The bottom part shows MT-TA^PBMC^ when adjusted for each of the established PE risk predictors. MT-TA^PBMC^ , MAP, Uterine artery PI (artUP) and sFlt-1/PlGF ratio were dichotomized according to Youden’s index: MT-TA^PBMC^ cut-off: 0.13, likelihood ratio (LHR): 3.2; MAP cut-off: 85.8, LHR: 2,1; artUP cut-off: 0.93, LHR: 1.9; sFlt-1/PlGF ratio cut-off: 9.3, LHR: 1.7

Finally, we tested if the RNAs validated to be DE in PE (i.e. MT-TA, MT-ND2, MT-CYB, CLU, PF4 and PPBP) could give relevant clinical information and predict the subsequent development of PE by evaluating their discriminatory properties when assessed at 22–24 weeks. As shown in Fig. 4A, based on ROC analyses the discriminatory properties of EV-derived RNAs was modest with AUCs between 0.6 and 0.7 when comparing PE (*n*=35) and normal pregnancies (*n*=35). As leukocytes may be a relevant, and readily obtainable, source and target of circulating EVs, we evaluated the most abundantly expressed target in leukocytes isolated in a previous study from this population [29]. The noncoding tRNA *MT-TA*. *MT-TA*^PBMC^ at 22–24 weeks gave a better discrimination for PE (Fig. 4A, AUC=0.82, *p*<0.001). As shown in Fig. 4B, leukocyte *MT-TA (MT-TA*^PBMC^*)* was significantly markedly lower in women who subsequently developed PE (*n*=38) compared to normal pregnancy (*n*=215) at weeks 22–24 and 36–38, but increased significantly during gestation. The same result was found for *MT-TA*^PBMC^ between PE and controls and prediction of PE when tested in a random-selected smaller control group (*n*=38) from the 215 controls (data not shown). We also measured *MT-TA* RNA directly in plasma, and in contrast, plasma levels of *MT-TA*, although detectable, were very low and showed no regulation in PE or temporal change during gestation (Fig. 4B). Furthermore, *MT-TA*^PBMC^ and *MT-TA* in EVs were significantly negatively correlated in women with PE (Fig. 4C), but not in normal pregnancy. This association was not observed at 36–38 weeks. Furthermore, in logistic regression using the cut-off determined by Youden’s index, *MT-TA*^PBMC^ gave a 15.3 times higher risk of developing PE (Fig. 4D), and this association was only modestly attenuated when adjusting for more established PE risk predictors.

### Plasma EV characterization using RNA-seq and qPCR data

**Fig. 5:**
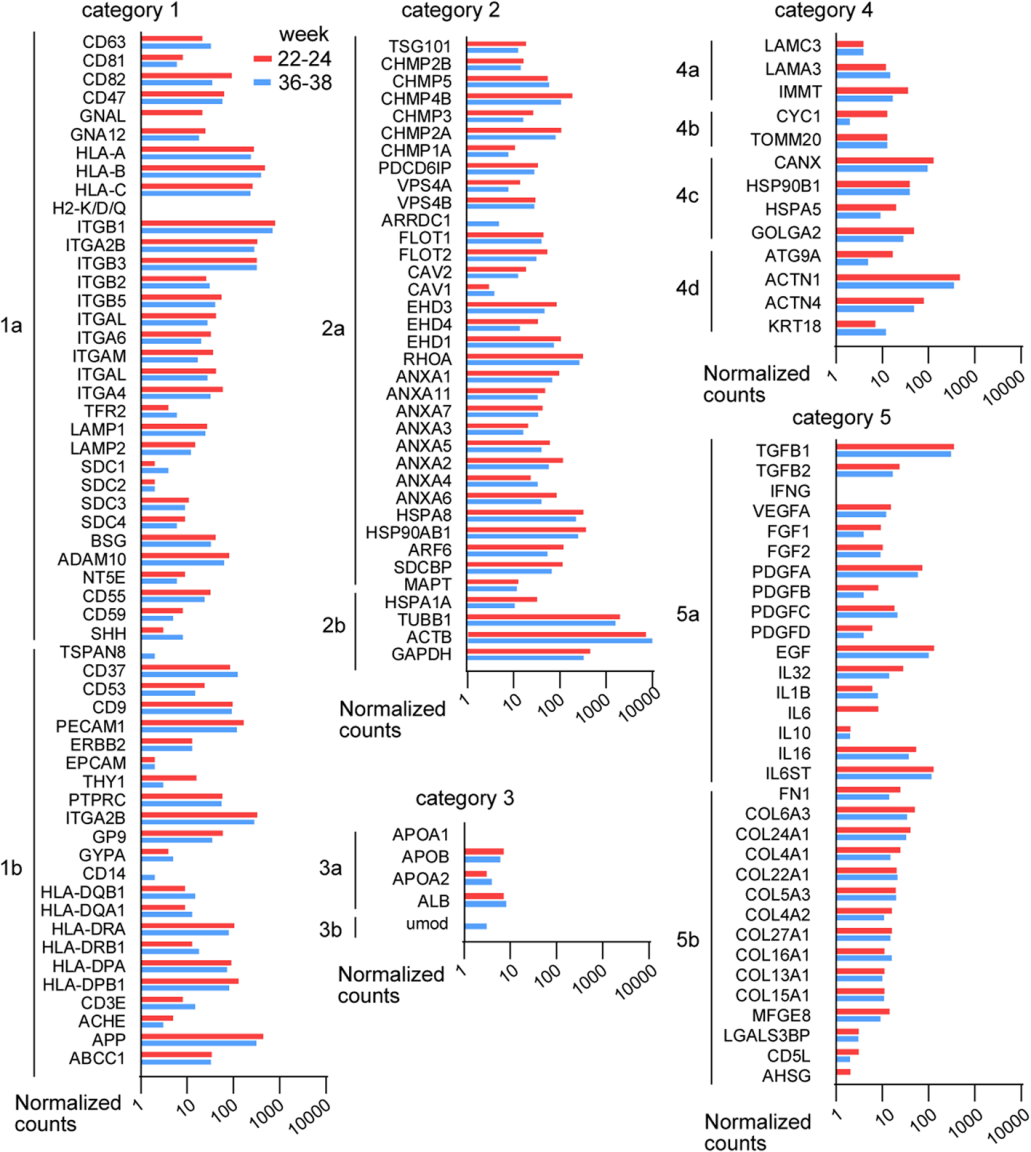
Average normalized counts for RNAs coding for protein used as EV markers (from MISEV2018 standards of EV research) in the controls from the RNA-seq analysis. *Category 1*, use for all EVs: Transmembrane or GPI-anchored proteins associated to plasma membrane and/or endosomes, 1a non-tissue specific, 1b tissue specific; *Category 2*, use for all EVs: Cytosolic proteins recovered in EVs, 2a with lipid or membrane protein-binding ability, 2b promiscuous incorporation in EVs (and possibly exomeres); *Category 3*, use for all EVs as purity control, major components of non-EV co-isolated structures: 3a lipoproteins (produced by liver, abundant in plasma, serum), 3b protein and protein/nucleic acid aggregates; *Category 4*, use for subtypes of EVs (e.g. large oncosomes, large EVs) and/or pathologic/atypical state, Transmembrane, lipid-bound and soluble proteins associated to other intracellular compartments than PM/endosomes: 4a nucleus, 4b mitochondrial, 4c secretory pathway (endoplasmic reticulum, Golgi apparatus), 4d others (autophagosomes, cytoskeleton); *Category 5*, use for functional component of EVs: need to determine the mode of association with EVs: 5a Cytokines and growth factors, 5b adhesion and extracellular matrix proteins

The proteins used to characterize EVs are presented in the standards for EV research MISEV2018 guideline [12]. When investigating RNA-seq data, the RNAs coding for these proteins (Fig. 5) were found in category 1; transmembrane or GPI-anchored proteins associated to plasma membrane and/or endosomes, especially MHC class I and some of the integrins highly expressed of the non-tissue-specific markers. In the tissue-specific markers, we found the leukocyte marker CD37, the endothelial cell marker PECAM1, platelet marker ITGA2B, some MHC class II markers, the neuron marker APP and CD9 (absent from NK, B and MSCs) relatively high expressed. In the category 2 markers, cytosolic proteins recovered in EVs, we found some of the lipid or membrane protein-binding ability CHMP* markers and accessory proteins relatively high expressed. For the promiscuous incorporation in EVs (and possibly exomeres) markers TUBB, ACTB and GAPDH were high expressed. In the category 3 markers, major components of non-EV co-isolated structures, we found all markers to be very low expressed.

Investigating markers specific for syncytiotrophoblast-derived EV, we found Flt-1 to be significantly increased during pregnancy, weeks 22–24 to 36–38 (expression level 12 to 32, adjusted *p*=0.05) in PE and elevated but not significantly increased in PE vs controls at weeks 36–38 (expression level 27 vs 11, adjusted *p*=0.11). The endoglin levels were low, increasing during pregnancy (expression level 2 to 9, adjusted *p*=0.06) in PE and elevated in PE at weeks 36–38 compared to controls (expression level 8 vs 1, adjusted *p*=0.04). In the validation cohort, we analyzed two of the miRNA from the cluster 19, mir-515-p and mir-518b, which are specific for the placental-derived EVs [39] and found them significantly increasing from weeks 22–24 to 36–38 in pregnancy, but with no significant differences expressed between PE and controls and not expressed in the EVs from the same participants 5 years after pregnancy (Additional file 1: Fig. S6).

### Pathway analysis using the regulated RNAs identified by RNA-seq analysis

We used g:Profiler to investigate pathways common to RNAs significant (FDR adjusted, <0.1) DE between PE and normal pregnancy at weeks 36–38 (Additional File 1: Fig. S2 and Additional file 2) and from weeks 22–24 to 36–38 in women who subsequently developed PE (Additional file 1: S3 and Additional file 2). Comparing PE and normal pregnancy at 36–38 weeks, we found very few pathways significantly upregulated and most were non-specific (e.g. transporter activity, organismal process, cilium). For RNAs significantly upregulated from 22–24 to 36–38 weeks during pregnancy in women who subsequently developed PE, KEGG identified cardiomyopathy, calcium signaling, ECM, cAMP, aldosterone and protein digestion and absorption as some of the major pathways. For the significantly downregulated pathways between PE and normal pregnancy and during PE development, platelet activation, mitochondrial, cellular senescence, infection, leukocyte transendothelial migration, ROS, integrin signaling, migration and vesicle biology were the major pathways. It is in the downregulated pathways that we find the most abundant RNAs.

### Circulating platelet activation markers and PlGF/sFlt-1 ratio

PE is known to have increased platelet activation and to further characterize the study population we measured P-selectin and PF4 in plasma at four time points during pregnancy. We found increasing P-selectin levels during pregnancy but no difference in the P-selectin or PF4 plasma levels between PE and controls (Additional file 1: Fig. S7). We also measured sFlt1/PlGF ratio as a marker of PE and placental dysfunction, showing higher levels in PE compared to controls at all time points (Additional file 1: Fig. S7).

## Discussion

In this study, we analyzed the transcriptomic signature of plasma EVs from women with PE, with a specific focus on mRNA and lncRNA. We found that (1) levels of platelet-derived and mitochondrial RNAs are abundant in circulating EVs; (2) expression of platelet-derived RNAs and mitochondrial RNAs are decreased and especially lncRNAs are increased, in EVs from PE compared to normal pregnancy as measured by RNA-seq; (3) decreased expression of mitochondrial RNAs *MT-TA*, *MT-ND2*, *MT-CYB* and platelet-derived RNAs *PPBP*, *PF4*, *CLU* in EVs from PE compared to normal pregnancy was confirmed by qPCR (*n*=60); and (4) decreased expression of the mitochondrial tRNA *MT-TA* in leukocytes at 22–24 weeks was strongly associated with the subsequent development of PE.

Evaluation of the most abundantly expressed RNAs in EVs revealed many that are considered a signature for blood platelets [37]. Indeed, platelets are likely a major source of EVs in plasma with some studies reporting they represent more than 25% of the content of circulating EVs [40, 41]. In addition, mitochondrial RNAs was abundantly expressed in EVs, which also could be derived from platelets as well as a range of cells in the maternal circulation or tissues such as the placenta [42]. Furthermore, the decrease in platelet-derived and mitochondrial RNAs in patients with PE was validated by qPCR (*n*=60) and importantly, they were decreased (at weeks 22–24) even before the diagnosis of PE. Many of the differentially upregulated RNAs in PE were novel lncRNA. Unfortunately, expression was so low that it was not feasible to measure by qPCR and validate using our current method.

In normal pregnancy a decrease in platelet count and an increase in platelet activation occurs, as reflected by levels of P-selectin in platelet-derived microparticles throughout gestation, supporting our result of increase in plasma P-selectin during pregnancy [43]. Plasma levels of platelet-derived molecules secreted from α-granules and adenosine secreted from dense granules are also higher in pregnancy, suggesting increased platelet activation and release of granule content [43]. It has been reported that syncytiotrophoblast-derived EVs contribute to thrombotic abnormalities in PE by activating platelets leading to its aggregation [44] linking coagulation and inflammation in these women [4]. Moreover, changes in platelet function, coagulation, and thrombotic factors are strongly associated with PE, and platelets from women with PE aggregate more easily and have increased mean platelet volume [45]. It has been suggested that, in PE, these maternal platelets with their small size can enter the intervillous space before uteroplacental blood flow is fully established [46] and adherent platelets have been detected on the villous surface of first trimester placental tissue [47]. Furthermore, activated maternal platelets, characterized by increase P-selectin, could promote endovascular trophoblast invasion in early pregnancy [48, 49] through secretion of growth factors, cytokines, and chemokines that may enhance trophoblast invasion. High levels of CCL5 and PF4, which may come from maternal platelets, have been found in placental explants [50]. Moreover, CCL5 enhances invasion of extravillous trophoblasts by binding to the chemokine receptor, CCR1, on extravillous trophoblasts [49]. However, early platelets and their cargo represent an important regulator of early human placental development. Accordingly, the content of placental syncytiotrophoblast-derived EVs can facilitate inflammatory responses and enhance endothelial cell dysfunction with effects on spiral artery remodeling and placentation [51] and we speculate that the lower levels of platelet RNA, including CCL5 and P-selectin in the EVs, may have similar effects. The invasion of extravillous trophoblasts is crucial for the remodeling of the spiral arteries [3]. We further speculate that the lower abundance of platelet-derived factors in EVs in women with PE could be due to excessive in vivo platelet activation, with diminishing content in secretory granules [52]. However, we observed no difference in platelet activation markers in plasma throughout pregnancy comparing normal and PE pregnancies. The difference could also be due to difference in the transcriptomic profile between the control and PE group [53]. It has been reported that older platelets have a lower RNA content [54]. In addition, it was recently reported variability of the transcriptome of platelets where the old platelets (smaller) have a more varied transcriptome than the young platelets (larger) [53, 55]. An upregulation of several transcripts involved in platelet activation and aggregation in the younger platelets, as prevalent in PE, has been reported [45, 56, 57]. Further, excess platelet activation, at the maternal-fetal interface, can provoke inflammasome activation in the placental trophoblasts and trigger formation of circulating platelet-monocyte aggregates, resulting in sterile inflammation of the placenta and systemic inflammatory response of the mother [46]. Importantly, whereas RNA-seq of EVs did not reveal any differences between PE and normal pregnancies at weeks 22–24, qPCR analyses (*n*=60) demonstrated lower levels of certain platelet-derived RNAs in PE at this time point, even before the diagnosis of PE, suggesting a potential pathogenic role of these processed in PE development.

Mitochondrial dysfunction has been implicated in the pathogenesis of PE [58]. Both intact mitochondria and mitochondrial components are found within EVs [59]. Their function depends on the donor and target cells [60, 61]. The reduced levels of mitochondrial RNA in PE could represent reduced reserves of mitochondrial components during times of increased mitochondrial respiration, such as during pregnancy [62]. Dysfunctional placental mitochondria are a major source of ROS production [58]. Recent studies have found decreased platelet mitochondrial membrane depolarization (mitochondrial distress and apoptosis) [63] and a decrease in all respiratory parameters in circulating platelets in PE [64]. Our finding that low levels of mitochondrial RNA in PE were inversely correlated with uterine artery PI, an established predictor of PE [38], may support the hypothesis that low (dysfunctional) mitochondrial RNA cargo in EVs could contribute to or reflect processes that are involved in PE progression. A recent study found the mitochondrial fusion protein mitofusin-2 increased in the circulation of PE and associated with uterine artery PI [65]. Mitochondrial fusion helps to reduce stress by mixing the contents of both damaged and undamaged mitochondria as a form of complementation potentially representing a compensatory mechanism against hypoxia and maternal endothelial dysfunction in PE [65]. These data are in line with our findings showing mitochondrial dysfunction reflected in the circulating EVs that could impact trophoblast invasion, and an increase in the fusion of damaged mitochondria to compensate and reduce the stress.

Evaluating the discriminatory abilities of the RNAs in EVs at weeks 22–24 revealed only modest prediction of PE. As circulating leukocytes may be a relevant source and/or target of EVs, our finding that *MT-TA* displayed a similar pattern of regulation in leukocytes as in EVs with lower levels in PE suggest that leukocytes may take up EVs from the circulation or that some of the EVs in the circulation are leukocyte-derived. Moreover, evidence show that placental EVs can interact with a multitude of maternal immune cells [4]. The negative correlation between EV and leukocyte-derived *MT-TA* at 22–24 weeks and opposite temporal pattern with increasing levels during gestation support such an interaction. Furthermore, the markedly stronger discriminatory power of leukocyte-derived *MT-TA* suggests that mitochondrial function and immune cells in PE deserve further attention. Placenta-derived EVs or other EVs can be recognized and internalized by immune cells and affect their function [66, 67]. Of note, platelet-monocyte aggregation is increased in women with PE [46]. Adhesion of platelets to monocytes induces the expression of sFlt-1 and may contribute to endothelial dysfunction and inflammation in PE. Mitochondria from stressed monocytes can also be a mediator of endothelial inflammation [68, 69].

The methods for isolating and characterization of EVs are of major importance. The commercial exoRneasy kit used in the current study has been compared with four other EV isolation methods and published in the ISEV journal in 2021 [70] and was shown to isolate the second most EV proteins, the least plasma proteins and contained a higher proportion of large EVs. Although there is no established “fingerprint” of RNA that reflect EVs, we found most mRNAs for proteins listed in the MISEV2018 guidelines [12] for validation of the EVs, and low expression for non-EV markers. An important technical issue investigating RNA-seq is that ribosomal RNA is typically depleted before the RNA-seq analysis, to enrich the detection of less abundant RNAs [71]. In our RNA-seq study of EVs, we did not deplete for ribosomal RNA which could explain the high level of ribosomal mitochondrial RNA. Further, using RNA-seq, we found an increased level of endoglin and Flt-1 in the PE samples, which are specific for syncytiotrophoblast-derived EVs. The abundant expression of markers specific for placental-derived EVs (i.e. mir-515-5p and mir-518b) during pregnancy, may also support the validity of the EVs [22]. However, the MISEV2018 guidelines state that more research is needed before specific recommendations can be made for using nucleic acids as specific markers of EVs or EV subtypes [12]. However, several RNAs have been found in EVs, and a variety of data have been shown on specific versus non-specific incorporation of RNAs into EVs or subtypes of EVs [72–75].

Despite an abundance of upregulated transcripts in women with PE at 36–38 weeks, KEGG pathway analysis did not reveal any dominant biological process, probably due in part to the presence of a large number of insufficiently characterized lncRNAs. Gene ontology analysis revealed mostly cellular components related to plasma membrane function, processes that include EV formation and processing. However, cilium is involved in different signaling pathways and has been proposed involved in PE development where dysfunctional cilia may lead to compromised migration, invasion, and endothelial remodeling of trophoblastic cells [76]. Conversely, a large number of KEGG pathways were identified when assessing transcripts that were upregulated in women who subsequently developed PE, including several related to the development of cardiomyopathy. Since PE is known to be associated with peripartum cardiomyopathy [77] and long-term cardiovascular risk [78], future studies are needed to investigate whether EV trafficking and signaling during pregnancy may promote cardiovascular disease [79].

The present study has several limitations. While the lncRNAs increased in PE may have important functions, most had low expression levels making it difficult to validate using our current RT-PCR/qPCR methodology. There are many methods for EV isolation [70, 80]. The exoRneasy kit, which we used in the current study, isolates large numbers of EV proteins and few non-EV proteins [70]. Compared to other methods, this kit isolates larger population of phenotypically larger cup-shaped EVs and may indicate that this kit favorably enriches for different types of EVs [70]. It might also give opportunities for different biomarker discovery, as stated in the comprehensive paper by Veerman et al. [70]. However, we did not include other methods to characterize the EVs and EV subpopulations, which is an important limitation of the present study. Moreover, samples for EV isolation was collected in tubes with sodium citrate additive and discussions are ongoing about EDTA additives may be better considering platelet activation [81, 82]. Also, post-collection methodology could influence EVs, and the FICOLL™ Hypaque™ gradient centrifugation that was used in the present study could have such properties. The change in instrument for isolating RNA for the PBMC is also a limitation of the study**.** Also, the controls in the validation study could have been better matched with regard to age and BMI, although these demographics were adjusted for in all statistical comparisons between groups as well as in the prediction analysis. Moreover, the women with PE in our study developed mostly late-onset PE. Finally, infections could influence the EV cargo. EVs can promote the replication and dissemination of viruses within the organism, through the dysregulation of their cargo and the modulation of the innate and adaptive immune response, but they can also promote the mitigation of viral infections [11]. The women included into the study did not have any symptoms of acute infections and none were using antibiotics or anti-viral agents; however, we cannot exclude any asymptomatic infections.

## Conclusions

This study identified abundant RNAs in plasma EVs and showed that many are differentially expressed in women with PE compared to normal pregnancy as well as in women who subsequently develop PE. In fact, whereas there were no differences in RNA-seq of EVs between PE and normal pregnancies at weeks 22–24, there was a marked difference in these transcripts at weeks 36–38, reflecting a marked change in RNA expression in EVs between these time points in PE but not in normal pregnancies. Both platelet-derived and mitochondrial RNA were highly expressed in EVs and decreased in EVs isolated from women with PE. In contrast, lncRNA subfraction was most significantly increased in PE. The mitochondrial RNA *MT-TA* in leukocytes may be a promising biomarker for early detection of PE.

### Supplementary Information

**Additional file 1:** **Table S1.** Alignment information on the RNA-seq analysis. **Table S2.** Primer sequences used in PCR reactions. **Figure S1.** Flow chart of inclusion and sample selection. **Figure S2.** Pathway analysis in PE vs. Controls at weeks 36-38. **Figure S3.** Pathway analysis in PE week 22-24 vs. PE week 36-38. **Figure S4.** Expressed RNAs (platelet derived) from week 22-24 to 36-38 in EVs from women developing PE. **Figure S5.** Expressed RNAs (mitochondrion) from week 22-24 to 36-38 in EVs from women developing PE. **Figure S6.** Expression of mi515-5p and mir518b in extracellular vesicles between women with PE and controls. **Figure S7.** Levels of P-selectin, PF4, sFlt1/PlGF ratio during pregnancy in women with PE and controls.

**Additional file 2.** G profiler analysis (significantly pathways) between women with PE vs. controls at 22-24 weeks and 36-38 weeks and between women with PE 22-24 weeks vs. 36-38 weeks.

## Data Availability

The datasets generated and/or analyzed during the current study are not publicly available due to ethical restrictions from the Regional Committee for Medical and Research Ethics in South‐East Norway but are available from the corresponding author on reasonable request.

## Abbreviations

CLU: Clusterin
lncRNA: Long noncoding RNA
MT-CYB: Mitochondrially encoded cytochrome b
MT-ND2: Mitochondrially encoded NADH dehydrogenase 2
MT-TA: Mitochondrially encoded tRNA alanine
PE: Preeclampsia
PF4: Platelet factor 4
PPBP: Pro-platelet basic protein

## References

[1] Palma C, Jellins J, Lai A, Salas A, Campos A, Sharma S, Duncombe G, Hyett J, Salomon C (2021). Extracellular vesicles and preeclampsia: current knowledge and future research directions. Subcell Biochem.

[2] Ghafourian M, Mahdavi R, Akbari Jonoush Z, Sadeghi M, Ghadiri N, Farzaneh M, Mousavi Salehi A (2022). The implications of exosomes in pregnancy: emerging as new diagnostic markers and therapeutics targets. Cell Commun Signal.

[3] Adam S, Elfeky O, Kinhal V, Dutta S, Lai A, Jayabalan N, Nuzhat Z, Palma C, Rice GE, Salomon C (2017). Review: fetal-maternal communication via extracellular vesicles - Implications for complications of pregnancies. Placenta.

[4] Nair S, Salomon C (2018). Extracellular vesicles and their immunomodulatory functions in pregnancy. Semin Immunopathol.

[5] Gonzalez-Quintero VH, Smarkusky LP, Jimenez JJ, Mauro LM, Jy W, Hortsman LL, O'Sullivan MJ, Ahn YS (2004). Elevated plasma endothelial microparticles: preeclampsia versus gestational hypertension. Am J Obstet Gynecol.

[6] Wang Z, Zhao G, Zeng M, Feng W, Liu J (2021). Overview of extracellular vesicles in the pathogenesis of preeclampsia. Biol Reprod.

[7] Paul N, Sultana Z, Fisher JJ, Maiti K, Smith R (2023). Extracellular vesicles- crucial players in human pregnancy. Placenta.

[8] Mincheva-Nilsson L (2021). Immunosuppressive protein signatures carried by syncytiotrophoblast-derived exosomes and their role in human pregnancy. Front Immunol.

[9] Rice GE, Scholz-Romero K, Sweeney E, Peiris H, Kobayashi M, Duncombe G, Mitchell MD, Salomon C (2015). The effect of glucose on the release and bioactivity of exosomes from first trimester trophoblast cells. J Clin Endocrinol Metab.

[10] Salomon C, Scholz-Romero K, Sarker S, Sweeney E, Kobayashi M, Correa P, Longo S, Duncombe G, Mitchell MD, Rice GE (2016). Gestational diabetes mellitus is associated with changes in the concentration and bioactivity of placenta-derived exosomes in maternal circulation across gestation. Diabetes.

[11] Martin C, Ligat G, Malnou CE. The Yin and the Yang of extracellular vesicles during viral infections. Biomed J 2023:100659. Online ahead of print.10.1016/j.bj.2023.10065937690583

[12] Thery C, Witwer KW, Aikawa E, Alcaraz MJ, Anderson JD, Andriantsitohaina R, Antoniou A, Arab T, Archer F, Atkin-Smith GK (2018). Minimal information for studies of extracellular vesicles 2018 (MISEV2018): a position statement of the International Society for Extracellular Vesicles and update of the MISEV2014 guidelines. J Extracell Vesicles.

[13] Murrieta-Coxca JM, Fuentes-Zacarias P, Ospina-Prieto S, Markert UR, Morales-Prieto DM (2022). Synergies of extracellular vesicles and microchimerism in promoting immunotolerance during pregnancy. Front Immunol.

[14] Dragovic RA, Southcombe JH, Tannetta DS, Redman CW, Sargent IL (2013). Multicolor flow cytometry and nanoparticle tracking analysis of extracellular vesicles in the plasma of normal pregnant and pre-eclamptic women. Biol Reprod.

[15] Gilani SI, Weissgerber TL, Garovic VD, Jayachandran M (2016). Preeclampsia and extracellular vesicles. Curr Hypertens Rep.

[16] Lok CA, Nieuwland R, Sturk A, Hau CM, Boer K, Vanbavel E, Vanderpost JA (2007). Microparticle-associated P-selectin reflects platelet activation in preeclampsia. Platelets.

[17] Knight M, Redman CW, Linton EA, Sargent IL (1998). Shedding of syncytiotrophoblast microvilli into the maternal circulation in pre-eclamptic pregnancies. Br J Obstet Gynaecol.

[18] Marques FK, Campos FM, Filho OA, Carvalho AT, Dusse LM, Gomes KB (2012). Circulating microparticles in severe preeclampsia. Clin Chim Acta.

[19] Rice TF, Donaldson B, Bouqueau M, Kampmann B, Holder B (2018). Macrophage- but not monocyte-derived extracellular vesicles induce placental pro-inflammatory responses. Placenta.

[20] Redman CW, Sargent IL (2007). Microparticles and immunomodulation in pregnancy and pre-eclampsia. Journal of reproductive immunology.

[21] Tong M, Chamley LW (2015). Placental extracellular vesicles and feto-maternal communication. Cold Spring Harb Perspect Med.

[22] Tannetta DS, Dragovic RA, Gardiner C, Redman CW, Sargent IL (2013). Characterisation of syncytiotrophoblast vesicles in normal pregnancy and pre-eclampsia: expression of Flt-1 and endoglin. PloS one.

[23] He X, He Y, Xi B, Zheng J, Zeng X, Cai Q, Ouyang Y, Wang C, Zhou X, Huang H (2013). LncRNAs expression in preeclampsia placenta reveals the potential role of LncRNAs contributing to preeclampsia pathogenesis. PloS One.

[24] Long W, Rui C, Song X, Dai X, Xue X, Lu Y, Shen R, Li J, Li J, Ding H (2016). Distinct expression profiles of lncRNAs between early-onset preeclampsia and preterm controls. Clin Chim Acta.

[25] McAninch D, Roberts CT, Bianco-Miotto T. Mechanistic Insight into Long Noncoding RNAs and the Placenta. Int J Mol sci. 2017;18(7):1371. 10.3390/ijms18071371 PMC5535864 28653993

[26] Gong RQ, Nuh AM, Cao HS, Ma M (2021). Roles of exosomes-derived lncRNAs in preeclampsia. Eur J Obstet Gynecol Reprod Biol.

[27] Sandrim VC, Luizon MR, Palei AC, Tanus-Santos JE, Cavalli RC (2016). Circulating microRNA expression profiles in pre-eclampsia: evidence of increased miR-885-5p levels. BJOG.

[28] Roland MC, Friis CM, Voldner N, Godang K, Bollerslev J, Haugen G, Henriksen T (2012). Fetal growth versus birthweight: the role of placenta versus other determinants. PloS one.

[29] Lekva T, Roland MCP, Estensen ME, Norwitz ER, Tilburgs T, Henriksen T, Bollerslev J, Normann KR, Magnus P, Olstad OK (2021). Dysregulated non-coding telomerase RNA component and associated exonuclease XRN1 in leucocytes from women developing preeclampsia-possible link to enhanced senescence. Scientific reports.

[30] Bushnell B: BBTools software package. 2014, https://sourceforge.net/projects/bbmap/.

[31] Pertea M, Kim D, Pertea GM, Leek JT, Salzberg SL (2016). Transcript-level expression analysis of RNA-seq experiments with HISAT StringTie and Ballgown. Nat Protoc.

[32] Liao Y, Smyth GK, Shi W (2014). featureCounts: an efficient general purpose program for assigning sequence reads to genomic features. Bioinformatics.

[33] Love MI, Huber W, Anders S (2014). Moderated estimation of fold change and dispersion for RNA-seq data with DESeq2. Genome Biol.

[34] Varet H, Brillet-Gueguen L, Coppee JY, Dillies MA (2016). SARTools: a DESeq2- and EdgeR-Based R Pipeline for comprehensive differential analysis of RNA-Seq Data. PloS One.

[35] Raudvere U, Kolberg L, Kuzmin I, Arak T, Adler P, Peterson H, Vilo J (2019). g:Profiler: a web server for functional enrichment analysis and conversions of gene lists (2019 update). Nucleic Acids Res.

[36] Lekva T, Michelsen AE, Aukrust P, Paasche Roland MC, Henriksen T, Bollerslev J, Ueland T (2017). CXC chemokine ligand 16 is increased in gestational diabetes mellitus and preeclampsia and associated with lipoproteins in gestational diabetes mellitus at 5 years follow-up. Diab Vasc Dis Res.

[37] Supernat A, Popeda M, Pastuszak K, Best MG, Gresner P, Veld SI, Siek B, Bednarz-Knoll N, Rondina MT, Stokowy T (2021). Transcriptomic landscape of blood platelets in healthy donors. Sci Rep.

[38] Khong SL, Kane SC, Brennecke SP, da Silva Costa F (2015). First-trimester uterine artery Doppler analysis in the prediction of later pregnancy complications. Dis Markers.

[39] Jaszczuk I, Winkler I, Koczkodaj D, Skrzypczak M, Filip A. The Role of Cluster C19MC in Pre-Eclampsia Development. Int J Mol Sci. 2022;23(22):13836. 10.3390/ijms232213836 PMC9699419 36430313

[40] Spakova T, Janockova J, Rosocha J. Characterization and Therapeutic Use of Extracellular Vesicles Derived from Platelets. Int J Mol Sci. 2021;22(18):9701. 10.3390/ijms22189701 PMC8468534 34575865

[41] Eustes AS, Dayal S. The Role of Platelet-Derived Extracellular Vesicles in Immune-Mediated Thrombosis. Int J Mol Sci. 2022;23(14):7837. 10.3390/ijms23147837 PMC9320310 35887184

[42] Amari L, Germain M (2021). Mitochondrial Extracellular Vesicles - Origins and Roles. Front Mol Neurosci.

[43] Forstner D, Guettler J, Gauster M. Changes in Maternal Platelet Physiology during Gestation and Their Interaction with Trophoblasts. Int J Mol Sci. 2021;22(19):10732. 10.3390/ijms221910732 PMC8509324 34639070

[44] Tannetta DS, Hunt K, Jones CI, Davidson N, Coxon CH, Ferguson D, Redman CW, Gibbins JM, Sargent IL, Tucker KL (2015). Syncytiotrophoblast extracellular vesicles from pre-eclampsia placentas differentially affect platelet function. PloS One.

[45] Walle M, Gelaw Y, Getu F, Asrie F, Getaneh Z (2022). Preeclampsia has an association with both platelet count and mean platelet volume: a systematic review and meta-analysis. PloS One.

[46] Moser G, Guettler J, Forstner D, Gauster M. Maternal Platelets-Friend or Foe of the Human Placenta? Int J Mol Sci. 2019;20(22):5639. 10.3390/ijms20225639 PMC6888633 31718032

[47] Blaschitz A, Siwetz M, Schlenke P, Gauster M (2015). Adhering maternal platelets can contribute to the cytokine and chemokine cocktail released by human first trimester villous placenta. Placenta.

[48] Sato Y, Fujiwara H, Konishi I (2010). Role of platelets in placentation. Med Mol Morphol.

[49] Sato Y (2020). Endovascular trophoblast and spiral artery remodeling. Mol Cell Endocrinol.

[50] Guettler J, Forstner D, Gauster M (2022). Maternal platelets at the first trimester maternal-placental interface - Small players with great impact on placenta development. Placenta.

[51] Cronqvist T, Tannetta D, Morgelin M, Belting M, Sargent I, Familari M, Hansson SR (2017). Syncytiotrophoblast derived extracellular vesicles transfer functional placental miRNAs to primary human endothelial cells. Sci Rep.

[52] Chaudhary PK, Kim S, Kim S. An Insight into Recent Advances on Platelet Function in Health and Disease. Int J Mol Sci. 2022;23(11):6022. 10.3390/ijms23116022 PMC9181192 35682700

[53] Thibord F, Johnson AD. Sources of variability in the human platelet transcriptome. Thromb Res. 2023;231(11):255–63. 10.1016/j.thromres.2023.06.009 PMC12442740 37357099

[54] Angenieux C, Maitre B, Eckly A, Lanza F, Gachet C, de la Salle H (2016). Time-dependent decay of mRNA and ribosomal RNA during platelet aging and its correlation with translation activity. PloS One.

[55] Clancy L, Beaulieu LM, Tanriverdi K, Freedman JE (2017). The role of RNA uptake in platelet heterogeneity. Thromb Haemost.

[56] Hille L, Lenz M, Vlachos A, Gruning B, Hein L, Neumann FJ, Nuhrenberg TG, Trenk D (2020). Ultrastructural, transcriptional, and functional differences between human reticulated and non-reticulated platelets. J Thromb Haemost.

[57] Bongiovanni D, Santamaria G, Klug M, Santovito D, Felicetta A, Hristov M, von Scheidt M, Aslani M, Cibella J, Weber C (2019). Transcriptome Analysis of Reticulated Platelets Reveals a Prothrombotic Profile. Thromb Haemost.

[58] Smith AN, Wang X, Thomas DG, Tatum RE, Booz GW, Cunningham MW (2021). The role of mitochondrial dysfunction in preeclampsia: causative factor or collateral damage?. Am J Hypertens.

[59] Sanz-Ros J, Mas-Bargues C, Romero-Garcia N, Huete-Acevedo J, Dromant M, Borras C. The potential use of mitochondrial extracellular vesicles as biomarkers or therapeutical tools. Int J Mol Sci. 2023;24(8):7005. 10.3390/ijms24087005 PMC10139054 37108168

[60] Liu D, Dong Z, Wang J, Tao Y, Sun X, Yao X (2020). The existence and function of mitochondrial component in extracellular vesicles. Mitochondrion.

[61] Lyamzaev KG, Zinovkin RA, Chernyak BV (2022). Extrusion of mitochondria: Garbage clearance or cell-cell communication signals?. J Cell Physiol.

[62] Holland OJ, Hickey AJR, Alvsaker A, Moran S, Hedges C, Chamley LW, Perkins AV (2017). Changes in mitochondrial respiration in the human placenta over gestation. Placenta.

[63] Kraemer BF, Hennis I, Karge A, Kraemer AK, Dreyer TF, Kiechle M, Kuschel B, Bronger H (2022). Platelet mitochondrial membrane depolarization reflects disease severity in patients with preeclampsia. Mol Med.

[64] Bina AM, Aburel OM, Avram VF, Lelcu T, Linta AV, Chiriac DV, Mocanu AG, Bernad E, Borza C, Craina ML (2022). Impairment of mitochondrial respiration in platelets and placentas: a pilot study in preeclamptic pregnancies. Mol Cell Biochem.

[65] Aydogan Mathyk B, Temel Yuksel I, Tayyar A, Aslan Cetin B, Tayyar AT, Koroglu N (2020). Maternal serum mitofusin-2 levels in patients with preeclampsia: the possible role of mitochondrial dysfunction in preeclampsia. J Matern Fetal Neonatal Med.

[66] Favaro RR, Murrieta-Coxca JM, Gutierrez-Samudio RN, Morales-Prieto DM, Markert UR (2021). Immunomodulatory properties of extracellular vesicles in the dialogue between placental and immune cells. Am J Reprod Immunol.

[67] Bai K, Lee CL, Liu X, Li J, Cao D, Zhang L, Hu D, Li H, Hou Y, Xu Y (2022). Human placental exosomes induce maternal systemic immune tolerance by reprogramming circulating monocytes. J Nanobiotechnol.

[68] Puhm F, Afonyushkin T, Resch U, Obermayer G, Rohde M, Penz T, Schuster M, Wagner G, Rendeiro AF, Melki I (2019). Mitochondria are a subset of extracellular vesicles released by activated monocytes and induce type I IFN and TNF responses in endothelial cells. Circ Res.

[69] Coly PM, Boulanger CM (2019). Extracellular Mitochondria and Vesicles. Circ Res.

[70] Veerman RE, Teeuwen L, Czarnewski P, Gucluler Akpinar G, Sandberg A, Cao X, Pernemalm M, Orre LM, Gabrielsson S, Eldh M (2021). Molecular evaluation of five different isolation methods for extracellular vesicles reveals different clinical applicability and subcellular origin. J Extracell Vesicles.

[71] Kissopoulou A, Jonasson J, Lindahl TL, Osman A (2013). Next generation sequencing analysis of human platelet PolyA+ mRNAs and rRNA-depleted total RNA. PloS One.

[72] Mateescu B, Kowal EJ, van Balkom BW, Bartel S, Bhattacharyya SN, Buzas EI, Buck AH, de Candia P, Chow FW, Das S (2017). Obstacles and opportunities in the functional analysis of extracellular vesicle RNA - an ISEV position paper. J Extracell Vesicles.

[73] Nolte-'t Hoen EN, Buermans HP, Waasdorp M, Stoorvogel W, Wauben MH (2012). t Hoen PA: Deep sequencing of RNA from immune cell-derived vesicles uncovers the selective incorporation of small non-coding RNA biotypes with potential regulatory functions. Nucleic Acids Res.

[74] van Balkom BW, Eisele AS, Pegtel DM, Bervoets S, Verhaar MC (2015). Quantitative and qualitative analysis of small RNAs in human endothelial cells and exosomes provides insights into localized RNA processing, degradation and sorting. J Extracell Vesicles.

[75] Li K, Rodosthenous RS, Kashanchi F, Gingeras T, Gould SJ, Kuo LS, Kurre P, Lee H, Leonard JN, Liu H, et al. Advances, challenges, and opportunities in extracellular RNA biology: insights from the NIH exRNA Strategic Workshop. JCI Insight. 2018;3(7):e98942. 10.1172/jci.insight.98942 PMC5928855 29618663

[76] Ritter A, Roth S, Kreis NN, Friemel A, Hoock SC, Steglich Souto A, Eichbaum C, Neuhoff A, Chen Q, Solbach C (2020). Primary cilia in trophoblastic cells: potential involvement in preeclampsia. Hypertension.

[77] Kuc A, Kubik D, Koscielecka K, Szymanek W, Mecik-Kronenberg T (2022). The relationship between peripartum cardiomyopathy and preeclampsia - pathogenesis diagnosis and management. J Multidiscip Healthc.

[78] Wu P, Haththotuwa R, Kwok CS, Babu A, Kotronias RA, Rushton C, Zaman A, Fryer AA, Kadam U, Chew-Graham CA et al. Preeclampsia and Future cardiovascular health: a systematic review and meta-analysis. Circ Cardiovasc Qual Outcomes. 2017;10(2):e003497. 10.1161/CIRCOUTCOMES.116.003497 28228456

[79] Pruthi D, Khankin EV, Blanton RM, Aronovitz M, Burke SD, McCurley A, Karumanchi SA, Jaffe IZ (2015). Exposure to experimental preeclampsia in mice enhances the vascular response to future injury. Hypertension.

[80] Gandham S, Su X, Wood J, Nocera AL, Alli SC, Milane L, Zimmerman A, Amiji M, Ivanov AR (2020). Technologies and standardization in research on extracellular vesicles. Trends Biotechnol.

[81] Wisgrill L, Lamm C, Hartmann J, Preissing F, Dragosits K, Bee A, Hell L, Thaler J, Ay C, Pabinger I (2016). Peripheral blood microvesicles secretion is influenced by storage time, temperature, and anticoagulants. Cytometry A.

[82] Almhanawi BH, Khalid B, Ibrahim TA, Tohit ERM (2017). A transmission electron microscopy study of anticoagulant-induced platelet vesiculation. Porto Biomed J.

