# Supplementary material for: Platelet and mitochondrial RNA is decreased in plasma-derived extracellular vesicles in women with preeclampsia—an exploratory study
Source: BMC Med. 2023 Nov 23;21:458. doi: 10.1186/s12916-023-03178-x (PMC10666366; doi:10.1186/s12916-023-03178-x)
Supplement: Supplementary file 1 — Additional file 1: Table S1. Alignment information on the RNA-seq analysis. Table S2. Primer sequences used in PCR reactions. Figure S1. Flow chart of inclusion and sample selection. Figure S2. Pathway analysis in PE vs. Controls at weeks 36-38. Figure S3. Pathway analysis in PE week 22-24 vs. PE week 36-38. Figure S4. Expressed RNAs (platelet derived) from week 22-24 to 36-38 in EVs from women developing PE. Figure S5. Expressed RNAs (mitochondrion) from week 22-24 to 36-38 in EVs from women developing PE. Figure S6. Expression of mi515-5p and mir518b in extracellular vesicles between women with PE and controls. Figure S7. Levels of P-selectin, PF4, sFlt1/PlGF ratio during pregnancy in women with PE and controls. [file 12916_2023_3178_MOESM1_ESM.doc]

Additional File 1

Table S1. Alignment information on the RNA-seq analysis

| **Sample** | **Weeks** | **Groups** | **Raw_read_pairs** | **Clean_read_pairs** | **Hisat2_overall_align_rate** | **FeatureCounts_unique_assigned_fragments** |
| --- | --- | --- | --- | --- | --- | --- |
| 1 | 22-24 | Control | 15994771 | 14763028 | 85,54 | 1048794 |
| 2 | 22-24 | Control | 22581014 | 20917084 | 87,54 | 1425874 |
| 3 | 22-24 | Control | 23597181 | 22431235 | 64,65 | 3697930 |
| 4 | 22-24 | Control | 17862158 | 17569698 | 82,34 | 1083605 |
| 5 | 22-24 | Control | 17518878 | 15952492 | 86,89 | 785980 |
| 6 | 22-24 | Control | 18270938 | 17543406 | 60,46 | 1298483 |
| 7 | 22-24 | Control | 20192065 | 19105863 | 77,18 | 3788030 |
| 1 | 36-38 | Control | 14433237 | 13649968 | 77,8 | 2925315 |
| 2 | 36-38 | Control | 23234083 | 21590829 | 86,93 | 2095866 |
| 3 | 36-38 | Control | 13436369 | 12818609 | 66,31 | 752639 |
| 4 | 36-38 | Control | 14487236 | 13718643 | 83,39 | 643561 |
| 5 | 36-38 | Control | 14770485 | 14420741 | 72,42 | 1121652 |
| 6 | 36-38 | Control | 13931349 | 13452146 | 64,36 | 1043733 |
| 7 | 36-38 | Control | 11863244 | 11228538 | 77,85 | 2367542 |
| 1 | 22-24 | PE | 17819559 | 17041519 | 73,71 | 1465907 |
| 2 | 22-24 | PE | 15065139 | 14582202 | 75,1 | 868774 |
| 3 | 22-24 | PE | 17202322 | 16602466 | 67,68 | 2733277 |
| 4 | 22-24 | PE | 19769086 | 18133723 | 84,35 | 2661636 |
| 5 | 22-24 | PE | 17086320 | 16341962 | 59,93 | 3737039 |
| 6 | 22-24 | PE | 13349936 | 12714136 | 64,87 | 1080608 |
| 7 | 22-24 | PE | 25468674 | 21799816 | 86,32 | 834482 |
| 1 | 36-38 | PE | 14941424 | 14482666 | 83,63 | 690727 |
| 2 | 36-38 | PE | 17848872 | 17408761 | 75,46 | 690411 |
| 3 | 36-38 | PE | 17718029 | 17067417 | 72,01 | 929305 |
| 4 | 36-38 | PE | 12707845 | 12290343 | 78,51 | 467038 |
| 5 | 36-38 | PE | 14742161 | 13907241 | 85,13 | 956143 |
| 6 | 36-38 | PE | 19663861 | 18407674 | 79,6 | 898792 |
| 7 | 36-38 | PE | 19557030 | 16392036 | 82,81 | 500563 |

Table S2. Primer sequences used in PCR reactions.

| **Genes** | **GenBank** | **Forward (5´to 3´)** | **Reverse (5´to 3´)** |
| --- | --- | --- | --- |
| PF4 | NM_002619.4  NM_001363352.1 | GCTGAAGCTGAAGAAGATGGG | TTCAGCGTGGCTATCAGTTG |
| PPBP | NM_002704.3 | TGGCGAAAGGCAAAGAGGAA | TCGACTTGGTTGCAATGGGT |
| MT-TA |  | GACTGCAAAACCCCACTCTG | AGGGCTTAGCTTAATTAAAGTGGC |
| MT-ND2 |  | CACAGAAGCTGCCATCAAGTA | CCGGAGAGTATATTGTTGAAGAG |
| MT-CYB |  | TCATCGACCTCCCCACCCCATC | CGTCTCGAGTGATGTGGGCGATT |
| CLU | NM_001831.3 | AGAAGAAAGAGGATGCCCTAAATG | GCCATCATGGTCTCATTGCAC |
| RPLP0 | NM_053275.4  NM_001002.4 | CAGATTGGCTACCCAACTGTT | GGAAGGTGTAATCCGTCTCCAC |
| ACTB | NM_001101.3 | AGGCACCAGGGCGTGAT | TCGTCCCAGTTGGTGACGAT |
| GAPDH | NM_001256799.1  NM_002046.4  NM_017008.3 | CCAAGGTCATCCATGACAACTT | AGGGGCCATCCACAGTCTT |


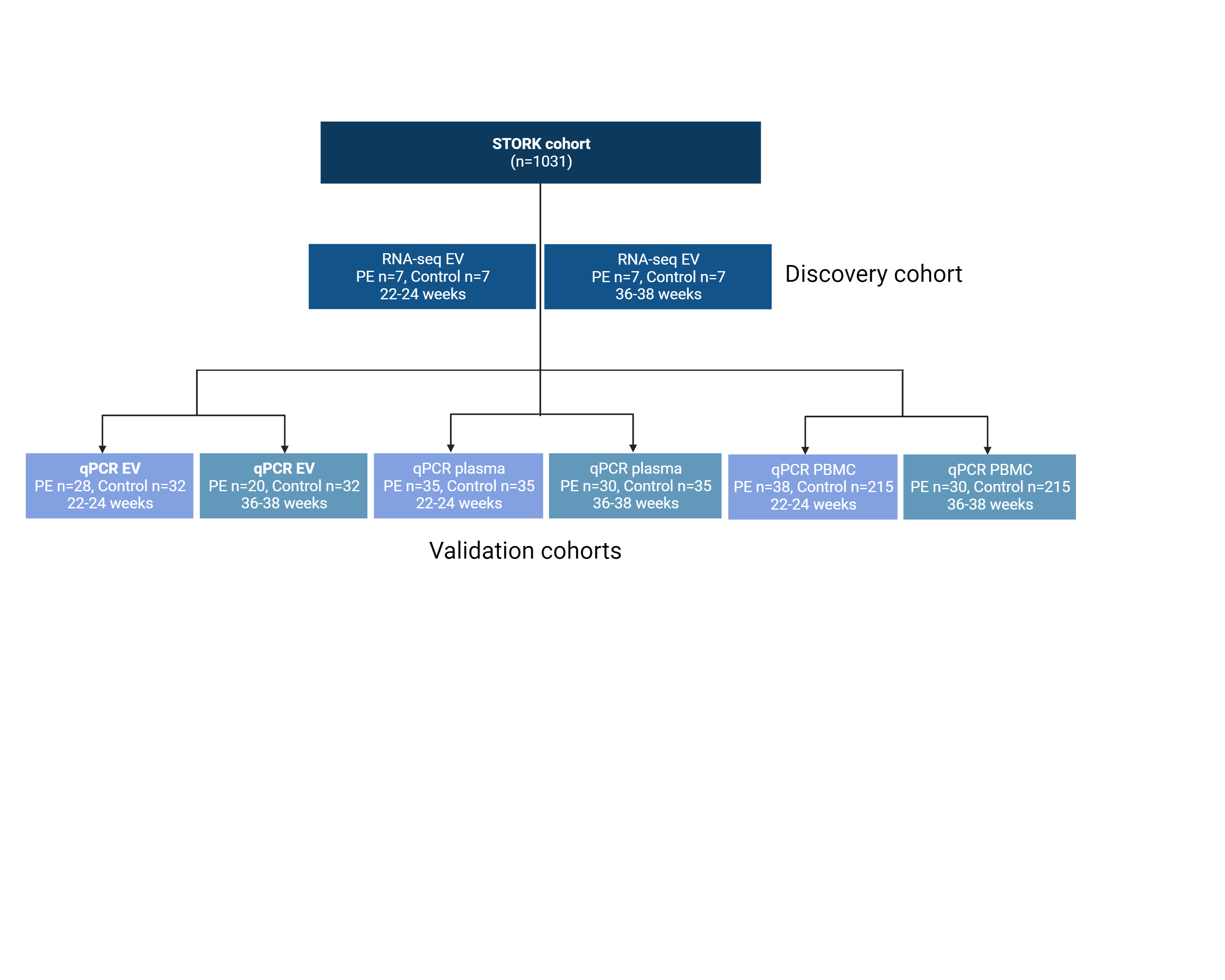


**Figure S1**. Flow chart of inclusion and sample selection.

Figure S2. Pathway analysis in PE vs. Controls at weeks 36-38. Analysis of differentially expressed genes for GO Biological Process (BP), Molecular Function (MF), Cellular Component (CC) term, KEGG and Reactome.


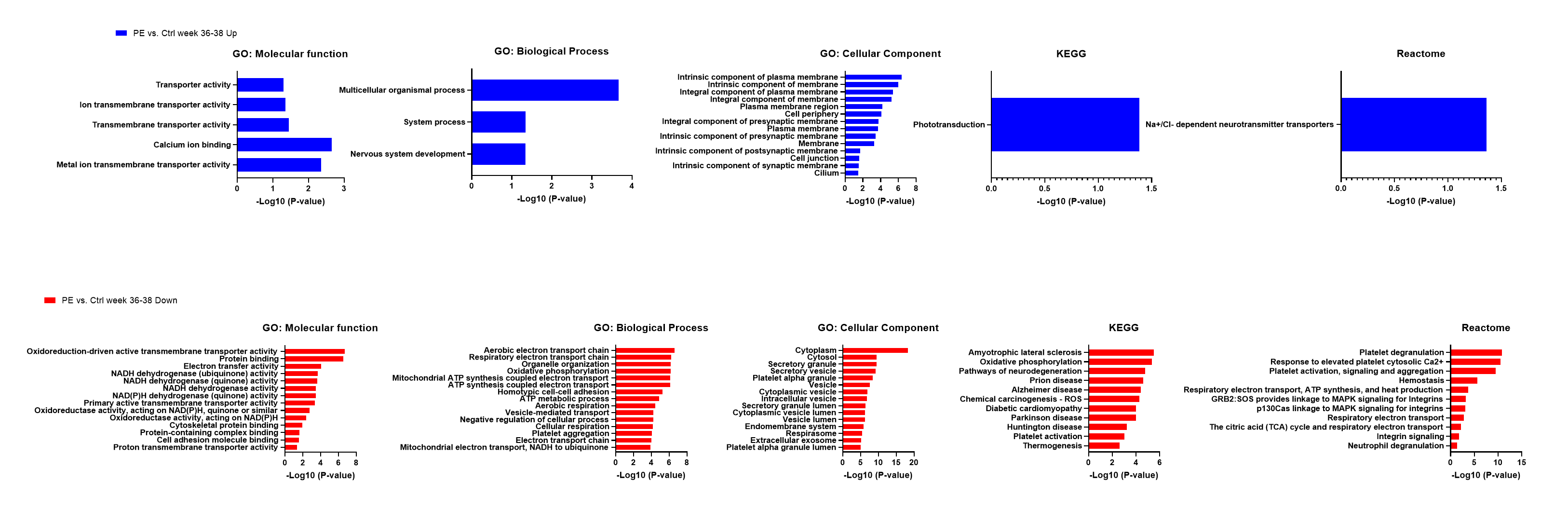


Figure S3. Pathway analysis in PE week 22-24 vs. PE week 36-38. Analysis of differentially expressed genes for GO Biological Process (BP), Molecular Function (MF), Cellular Component (CC) term, KEGG and Reactome.


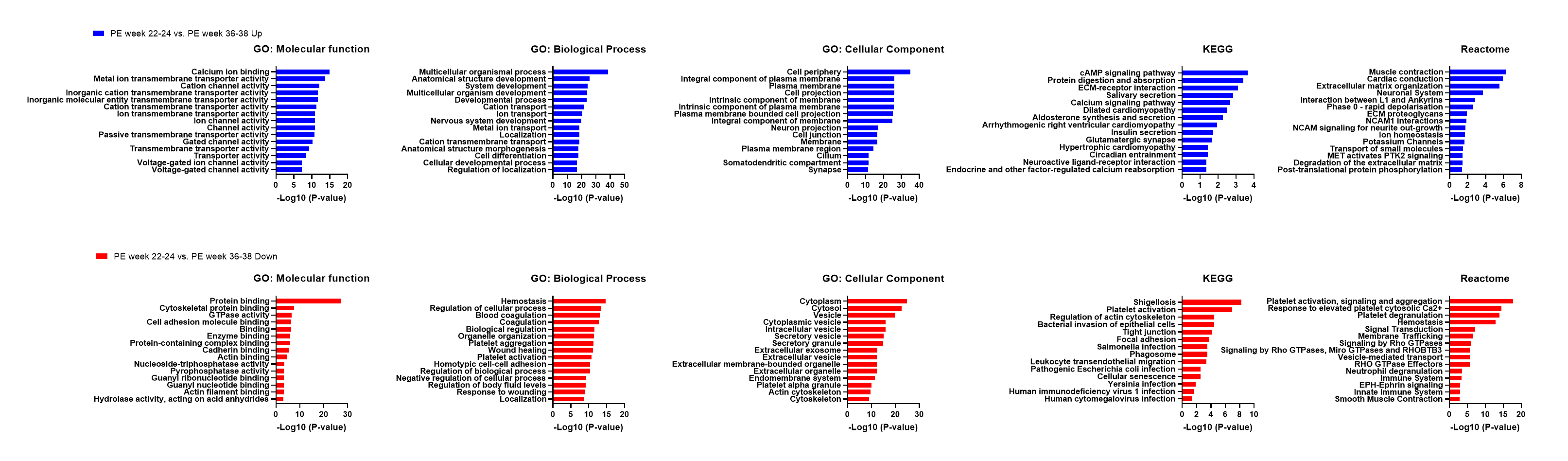


Figure S4. Expressed RNAs (platelet derived) from week 22-24 to 36-38 in EVs from women developing PE. Normalized counts and adjusted p-values are presented.


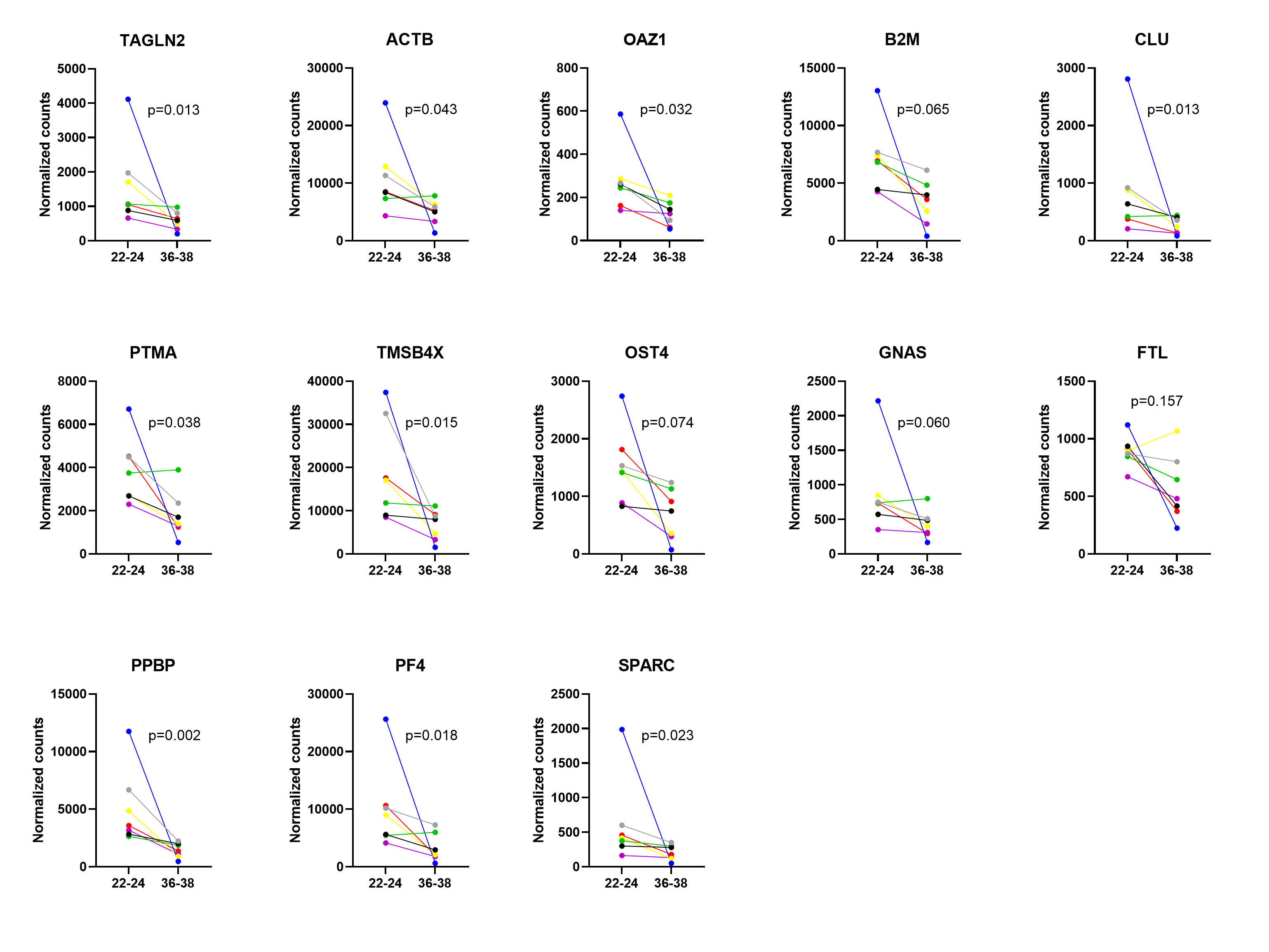


Figure S5. Expressed RNAs (mitochondrion) from week 22-24 to 36-38 in EVs from women developing PE. Normalized counts and adjusted p-values are presented.


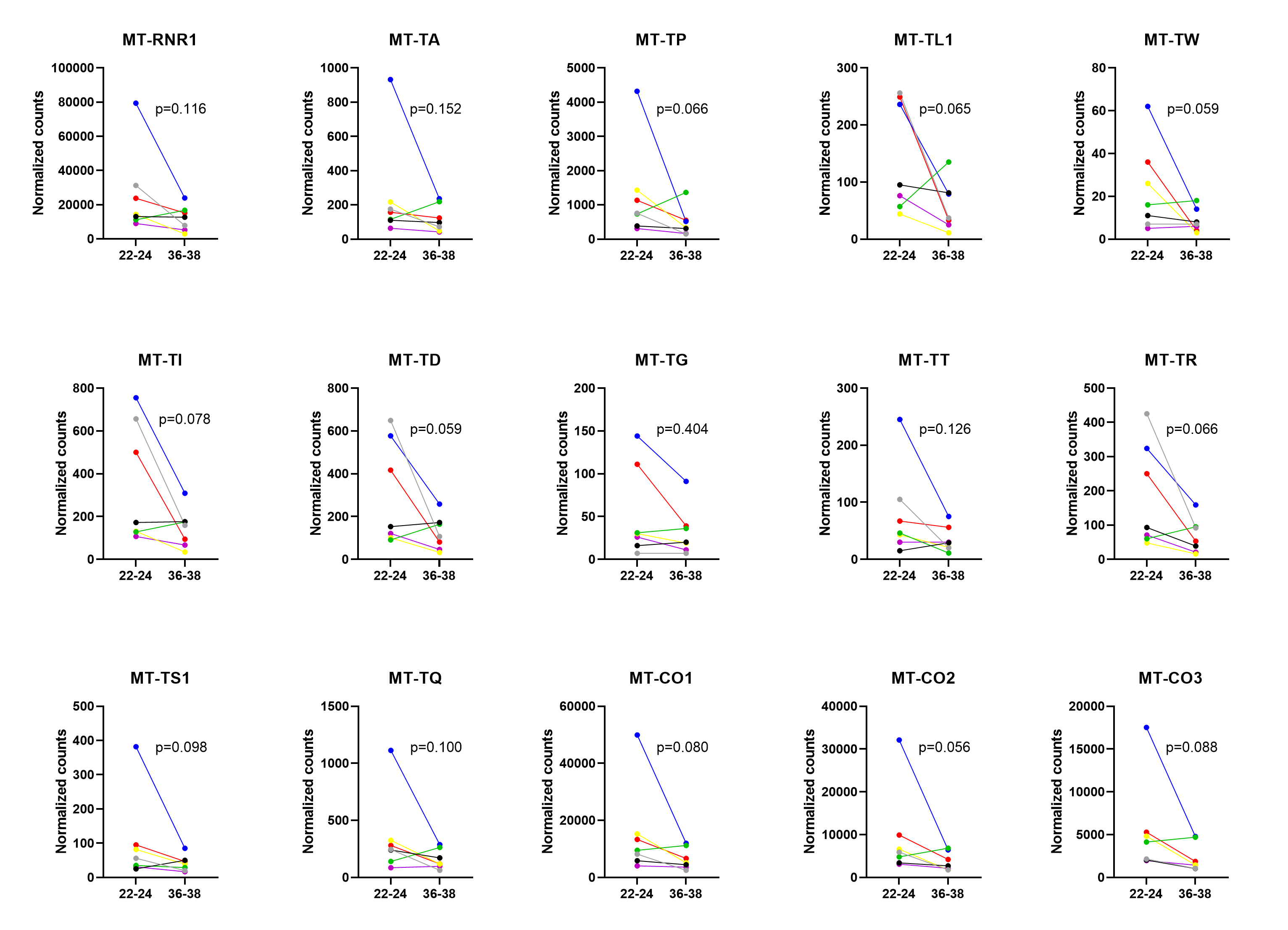


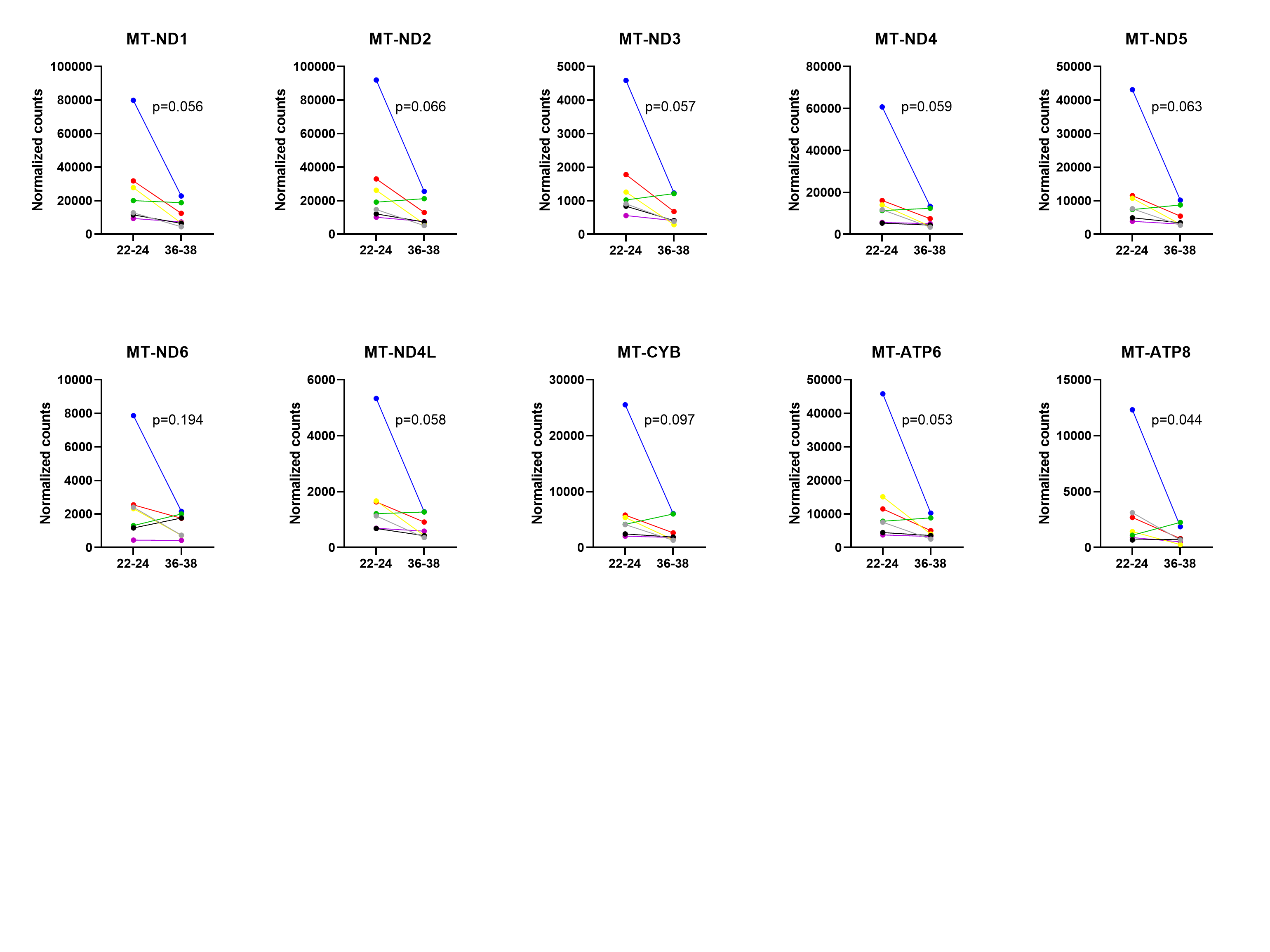


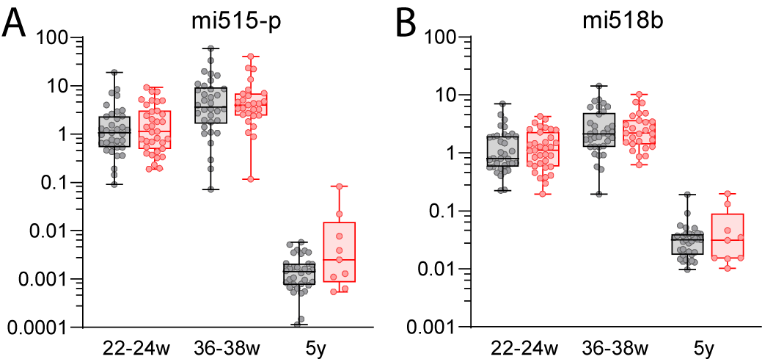


**Figure S6.** Expression of (**A)** *mi515-5p and* (**B)** *mir518b* in extracellular vesicles between PE (n=35) (red) and controls (n=35) (black) at different timepoints during pregnancy and at 5 years follow-up.


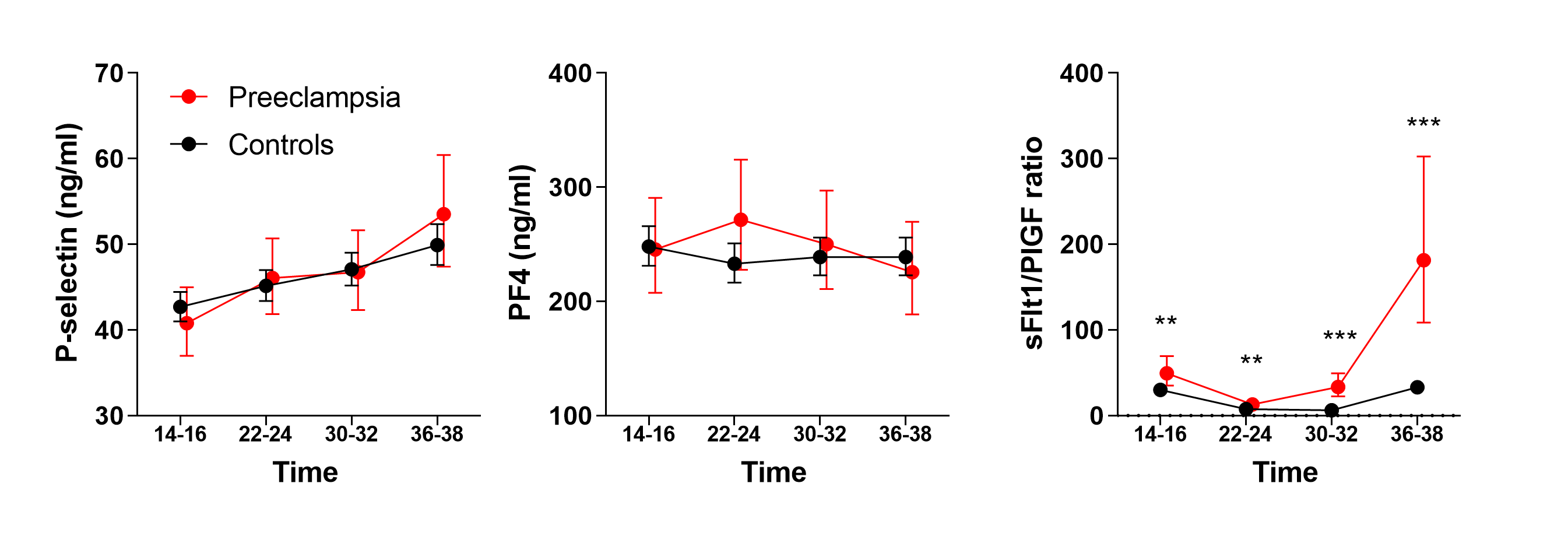


**Figure S7**. Levels of P-selectin, PF4, sFlt1/PlGF ratio during pregnancy in PE (n=38) (red) and controls (n=215) (black), adjusted for age. Data presented as estimated marginal means (95% CI). **p<0.01, ***p<0.001 comparing groups at the same timepoi
